# Catalytic Enantioselective Addition of Organozirconium Reagents to Aldehydes

**DOI:** 10.3390/molecules23040961

**Published:** 2018-04-20

**Authors:** Ricard Solà, Marcos Veguillas, María José González-Soria, Nicholas Carter, M. Angeles Fernández-Ibáñez, Beatriz Maciá

**Affiliations:** 1 Division of Chemistry & Environmental Science, Manchester Metropolitan University, Oxford Road, Manchester M1 5GD, UK; rsolamestres@gmail.com (R.S.); marcos_veguillas@yahoo.es (M.V.); mariajo.gonzalez.s@gmail.com (M.J.G.-S.); ncarter1@sheffield.ac.uk (N.C.); 2 Van ’t Hoff Institute for Molecular Sciences, Science Park 904, Amsterdam 1090 GS, The Netherlands

**Keywords:** alkenes, asymmetric catalysis, titanium, addition to aldehydes, Schwartz reagent

## Abstract

A catalytic enantioselective addition reaction of alkylzirconium species to aromatic aldehydes is reported. The reaction, facilitated by a chiral nonracemic diol ligand complex with Ti(O*^i^*Pr)_4_, proceeds under mild and convenient conditions, and no premade organometallic reagents are required since the alkylzirconium nucleophiles are generated in situ by hydrozirconation of alkenes with the Schwartz reagent. The methodology is compatible with functionalized nucleophiles and a broad range of aromatic aldehydes.

## 1. Introduction

Chiral alcohol-containing molecules are recurrent, high-value targets in the pharmaceutical, agricultural, and material science sectors, amongst others; the development of efficient methods for their construction remains a high priority in organic synthesis [1].

The catalytic enantioselective 1,2-addition reaction of organometallic reagents to carbonyl compounds is one of the most efficient approaches to chiral alcohols. This transformation has been extensively studied with dialkylzinc [2,3,4,5,6,7,8,9,10,11,12,13,14,15,16,17] and trialkylaluminium [18,19,20] reagents; more recently, excellent results with Grignard [21,22,23,24,25,26,27,28,29,30,31,32,33,34,35] and organolithium [36,37,38] reagents have also been reported. The high reactivity and sometimes pyrophoric character of these premade, non-stabilized organometallic nucleophiles, however, restricts the implementation of these methodologies in industrial processes and large-scale reactions [39]. Other complicating factors are the frequent requirement for cryogenic temperatures (necessary in order to obtain high levels of enantioselectivity but often prohibitively expensive at large scale) and incompatibilities with several functional groups [40,41]. The use of less reactive nucleophiles circumvents some of these issues. Organozirconium reagents [42,43,44,45,46,47,48] are relatively inert toward carbonyl compounds [49], but the use of catalysts or a stoichiometric mediator [50,51,52,53,54,55,56,57,58,59,60] enables the nucleophilic attack and subsequent carbon-carbon bond formation. Thus, in the presence of Ag(I), ZnBr_2_, or Me_2_Zn, organozirconium reagents can readily be added to aldehydes [61,62,63,64,65,66,67,68], ketones [69,70], and also enones [71,72,73,74], epoxides [75], and isocyanates [76], although enantioselective protocols have been rare so far [77,78,79,80,81,82,83,84,85,86,87,88,89,90,91,92]. In 1994, Wipf reported [63,64] a high-yielding protocol for the in situ transmetalation of alkenylzirconocenes to alkenylzinc species with stoichiometric amounts of Me_2_Zn, and succeeded in developing a catalytic asymmetric methodology for their subsequent additions to aldehydes [93,94]. A similar strategy was adopted by Walsh et al. for the addition of alkenylzirconocenes to ketones catalysed by a bis-(sulphonamide) diol ligand in the presence of stoichiometric Ti(O*^i^*Pr)_4_ [95]. We are not aware, however, of any successful report addressing the catalytic asymmetric addition of alkylzirconocene nucleophiles to carbonyls [14,31,96].

The use of alkylzirconocene reagents in synthesis is greatly facilitated by their ready accessibility via (in situ) hydrozirconation of alkenes using Schwartz reagent [97,98,99] (Cp_2_ZrHCl). This offers two key advantages: (i) alkenes, as starting materials, are inexpensive, abundant, and easy to handle [100]; (ii) hydrozirconation reaction conditions are compatible with many functional groups [101]. 

Here, we report the first enantioselective catalytic 1,2-addition of alkylzirconium reagents to aldehydes, based on a titanium-Ar-BINMOL complex. This methodology affords high levels of enantioselectivity at industrially relevant temperatures and the reaction tolerates functional groups that are not compatible with other organometallic reagents.

## 2. Results and Discussion

Provided with both axial and tetrahedral chirality, 1,1-binaphthalene-2-α-arylmethan-2-ols (Ar-BINMOLs)—developed by Kiyooka, Lai, and Xu [102,103,104,105]—have recently emerged as very efficient ligands for the titanium-assisted catalytic addition of organometallic reagents to carbonyl compounds [21,23,30,36,37]. Here, we started our investigations by evaluating the use of the very versatile Ph-BINMOL [23,102,103,104,105] ligand in the addition of 1-hexene to benzaldehyde (Table 1). Following known procedures [97,98,99], the treatment of 1-hexene with 2.0 eq. of Schwartz reagent (Cp_2_ZrHCl) provided the corresponding organozirconium reagent, which was then added to a solution of benzaldehyde (1.0 eq., 0.125 M) and Ph-BINMOL (20 mol%) in DCM at RT (Table 1). As expected, very low conversion to the desired alcohol **3aa** was observed (13%, entry 1). Under similar conditions (0.125 M in benzaldehyde), the reaction was attempted in the presence of 2.5–2.8 eq. of various additives (AgOTs, Ti(O*^i^*Pr)_4_, TiCl(O*^i^*Pr)_3_, CuI, and Et_2_Zn) in DCM at RT. No conversion was observed except in the presence of 2.0 eq. of Et_2_Zn (19% conversion to racemic **3aa**; entry 2). In accordance with Srebnik’s observations [62], more concentrated reaction conditions (0.5 M benzaldehyde in DCM) provided higher conversion to the desired product **3aa** (44%, entry 3), although the enantioselectivity of the process remained zero. 

An extensive screening of zinc additives revealed that the use of ZnBr_2_ (0.5 eq.) in combination with Ti(O*^i^*Pr)_4_ (1.5 eq.) provides the desired alcohol **3aa** in 83% isolated yield and 80% *ee*, using only 1.4 eq. of the alkene and 1.2 eq. of the Schwartz reagent, in DCM (0.5 M benzaldehyde) at RT (entry 4). It is important to mention that the reaction proved to be very sensitive to the concentration and no conversion was observed under more diluted conditions (0.11 M benzaldehyde in DCM, entry 5). 

Working at the preferred 0.5 M concentration of substrate in DCM, variation of the titanium source (TiCl(O*^i^*Pr)_3_ instead of Ti(O*^i^*Pr)_4_), however, resulted in increased reduction of the starting material to phenylmethanol, whilst the desired product **3aa** was obtained in a racemic form (entry 6). Co-solvents—*tert*-butylmethyl ether, THF, toluene, and diethyl ether—were also assayed in combination with DCM, which we found to be optimal for the hydrozirconation step; all attempts provided lower conversion and enantioselectivity than the use of DCM alone.

Changes in the titanium loading (entries 7–8) or the amount of ZnBr_2_ (entries 9–10), only afforded increased amounts of the undesired reduced product and lower enantioselectivities. To our surprise, when the reaction was carried out at lower temperature (0 °C, overnight), lower enantioselectivity was observed (35% *ee*, entry 11), whilst higher temperatures (35 °C) provided slightly higher enantioselectivity than RT (82% *ee*, compare entries 12 and 4), but lower conversion (51%). By way of comparison, the reaction was assessed using (*R*)-BINOL (20 mol %) as ligand; 9% conversion to the desired product **3aa** was obtained in 56% *ee* (entry 13). 

Lowering the amounts of the Schwartz reagent and the alkene provided higher enantioselectivity (90%) but lower conversion to the desired **3aa**, due to a substantial increase in the reduction by-product (entry 14). Fortunately, improved results were obtained with increased amounts of Schwartz reagent and the alkene, and, after fine adjustments, 99% conversion and 93% *ee* could be reached in 5 h when 2.0 eq. of Schwartz reagent were used in combination with 2.2 eq. of alkene in DCM at 35 °C (entry 15).

Regarding the mechanism of the addition reaction, a number of pathways are possible. The transmetallation of the organozirconium reagent with ZnBr_2_ [106,107,108], followed by second transmetallation with the appropriate organotitanium species is a very plausible route [97,98,99]. However, the activation of aldehydes by complexation with zinc halides [109] is a well-known process that cannot be discarded at this stage of our investigations. It is worth pointing out the versatility of Ar-BINMOL ligands, in particular the simple and readily available Ph-BINMOL, which is able to catalyse the carbonyl addition of a broad spectrum of organometallic reagents, including organozinc [110], organomagnesium [21,23,30], organolithium [36,37,38], organoaluminum [20], organotitanium [111], and, now, organozirconium reagents. As far as we know, this catalytic system allows the broadest variety of organometallic reagents in enantioselective 1,2-addition to carbonyl groups.

**Figure 1:**
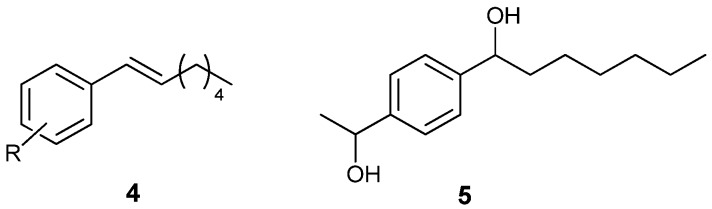
By-products of the reaction.

With the optimised conditions in hand, we tested the scope of the reaction with different aromatic aldehydes (Table 2). Thus, the reaction of 1-hexene (**2a**) with *p*-tolualdehyde afforded product **3ba** with good yield (74%) and excellent enantioselectivity (91%, entry 1). In the case of *m*- and *o*-tolualdehyde (entries 2 and 3), where the methyl substituent in the aromatic ring is closer to the reactive site, higher percentages of the corresponding aryl methanol (reduction of the aldehyde) and dehydration products **4** (Figure 1) were obtained, as well as lower enantioselectivity (89% and 76%, respectively); this is probably due to increased steric hindrance close to the carbonyl group. The reaction with *p*-bromo and *p*-chlorobenzaldehyde afforded moderated yields (56% and 59%) and excellent enantioselectivities (91% and 90%, entries 4 and 5, respectively). The use of *p*-acetylbenzaldehyde as starting material (entry 6), provided the corresponding alcohol **3ga** in excellent enantioselectivity (94%) but lower yield (32%). This is probably due to the reduction of the acetyl group by β-hydride transfer from the organometallic reagent (by-product 5, Figure 1). Gratifyingly, the methodology proved to be compatible with other functional groups such as *p*-CN (entry 7) and *p*-CF_3_ (entry 8), leading to good yield (55–58%) and high enantioselectivity (87% *ee*). Unfortunately, aliphatic and α,β-unsaturated aldehydes gave very low conversions under these reaction conditions. 

Next, we tested the scope of the reaction with different alkenes (Table 3). Thus, the reaction of 4-phenyl-1-butene (**2b**) with benzaldehyde (**1a**) provided the corresponding alcohol **3ab** in excellent yield (93%) and good enantioselectivity (77% *ee*, entry 1). The methodology is also compatible with functionalised alkenes. The reaction of benzaldehyde with 4-[(*tert*-butyldimethylsilyl)oxy]-1-butene (**2c**) led to the desired alcohol **3ac** in moderate yield (42%) but good enantioselectivity (88% *ee*, entry 2). Similar results were obtained when 4-halo-1-butenes **2d** and **2e** were used as nucleophiles (entries 3 and 4), providing **3ad** and **3ae** in moderate yields and 85 and 74% *ee*, respectively. The use of 5-bromopent-1-ene (**2f**) provided **3af** in 31% yield and 81% *ee*.

As an application of this methodology, product **3ad** was transformed into its corresponding tetrahydropyran adduct **6**. Tetrahydropyran rings are very important structural moieties, which are present in a large variety of natural products such as polyether antibiotics and marine macrocycles [112,113,114,115,116]. Additionally, they are also employed in the perfume industry or as flavouring ingredients in the food industry [117]. 

**Scheme 1:**
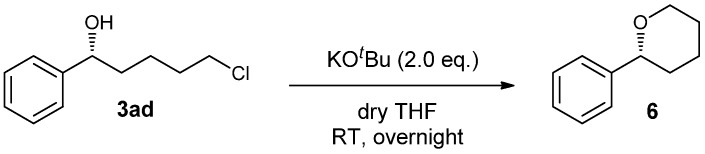
Formation of the chiral tetrahydropyran **6** from chiral chloroalcohol **3ad**.

Thus, following a straightforward procedure [118], alcohol **3ad** was dissolved in dry THF and treated with 2 eq. of KO*^t^*Bu at RT. Tetrahydropyran **6** was obtained in 84% yield and 85% *ee* after purification by column chromatography (Scheme 1). It is worth pointing out that no racemization occurs during the cyclization [119]. This strategy constitutes a novel and straightforward method for the synthesis of chiral tetrahydropyran derivatives via an enantioselective 1,2-addition of an alkene to a carbonyl followed by an intramolecular SN_2_ reaction.

## 3. Materials and Methods 

General procedure for the catalytic enantioselective 1,2-addition of alkenes to aldehydes: To a stirred suspension of Cp_2_ZrHCl (77 mg, 0.3 mmol, 2.0 eq.) in dry DCM (0.3 mL) at RT, the corresponding alkene (0.33 mmol, 2.2 eq.) was added dropwise and the solution was stirred at RT for 30 min. The mixture turned into a clear yellow solution, which indicated the successful formation of the organozirconium reagent. Next, flamed-dried ZnBr_2_ (0.075 mmol, 0.5 eq.) was added into the solution and the mixture was stirred at RT for 2 min. Subsequently, a solution of Ti(O*^i^*Pr)_4_ (0.225 mmol, 1.5 eq.) and (*R*_a_,*S*)-Ph-BINMOL (20 mol %) in dry DCM (0.1 mL) was added and stirred for further 2 min at RT. Finally, the aldehyde (0.15 mmol) was added and the solution was stirred at 35 °C for 3–18 h (reaction was monitored by TLC). (Note that liquid aldehydes were previously distilled before its addition whilst solid aldehydes were dissolved in dry DCM (0.1 or 0.2 mL depending on its solubility) and added to the solution.) The reaction was quenched by the addition of water (1 mL). The layers were separated, and the aqueous layer was extracted with Et_2_O (3 × 10 mL). The combined organic layers were dried with anhydrous MgSO_4_, filtered, and concentrated under vacuum. The crude reaction product was purified by flash silica gel chromatography.

## 4. Conclusions

In conclusion, we have developed a new and efficient procedure for the titanium-assisted catalytic asymmetric addition of alkylzirconium reagents to aromatic aldehydes, based on the use of a readily available Ar-BINMOL ligand and ZnBr_2_. The alkylzirconium nucleophiles are generated in situ by hydrozirconation of alkenes with Schwartz reagent, thus avoiding the use of premade organometallic reagents. The reaction—which proceeds under mild conditions and industrially relevant temperatures—allows the synthesis of the corresponding chiral secondary alcohols in moderate to good yields (32–93%) and good to excellent enantioselectivities (76–91% *ee*). It is worth mentioning that the methodology is compatible with the presence of several functional groups in both the aldehyde (including halogens, ketone, cyano, and trifluoromethyl) and the alkene (including halogens and TBS protected alcohol). The usefulness of this novel method has been demonstrated with the enantioselective synthesis of a chiral tetrahydropyran by a subsequent intramolecular cyclization on a functionalised addition product.

## Figures and Tables

**Table 1:**
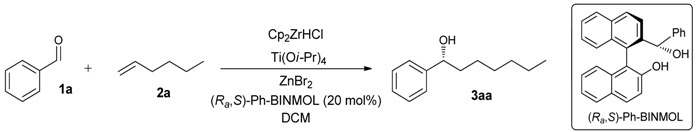
Optimisation of reaction conditions for the addition of 1-hexene (**2a**) to benzaldehyde (**1a**) ^a^.

Entry	Cp_2_ZrHCl (eq.)	1-hexene (eq.)	Ti(O*^i^*Pr)_4_ (eq.)	ZnBr_2_ (eq.)	Conv. (%) ^b^	Undesired Phenylmethanol (%) ^b^	*ee* (%) ^c^
1 ^d^	2	2	-	-	13	0	0
2 ^d^	2	2	-	- ^e^	19	0	0
3	2	2	-	- ^e^	44	0.	
4	1.2	1.4	1.5	0.5	n.d. (83) ^f^	10	80
5 ^g^	1.2	1.4	1.5	0.5	1	n.d.	0
6	1.2	1.4	- ^h^	0.5	22	78	0
7	1.2	1.4	1.0	0.5	43	57	35
8	1.2	1.4	2.0	0.5	5	89	62
9	1.2	1.4	1.5	0.2	18	73	80
10	1.2	1.4	1.5	0.7	20	74	66
11 ^i^	1.2	1.4	1.5	0.5	6	67	35
12 ^j^	1.2	1.4	1.5	0.5	51	36	82
13 ^k^	1.2	1.4	1.5	0.5	9	83	56
14	1.0	1.2	1.5	0.5	11	59	90
15 ^j^	2.0	2.2	1.5	0.5	99 (87) ^f^	5	93 (*R*) ^l^

^a^ Reaction conditions: **1a** (0.15 mmol, 1.0 eq.), (*R*_a_,*S*)-Ph-BINMOL (0.2 eq.), Ti(O*^i^*Pr)_4_ (1.5 eq.), DCM (0.5 M), room temperature, overnight. ^b^ Determined by CG-MS. ^c^ Determined by Chiral GC (see supplementary material for further details). ^d^ 0.125 M in benzaldehyde. ^e^ Reaction carried out with Et_2_Zn instead of ZnBr_2_. ^f^ Isolated yield after flash chromatography. ^g^ 0.11 M **1a** in DCM. ^h^ Reaction carried out with 1.5 eq. of TiCl(O*^i^*Pr)_3_ instead of Ti(O*^i^*Pr)_4_. ^i^ Reaction carried out at 0 °C. ^j^ Reaction carried out at 35 °C. ^k^ (*R*)-BINOL (20 mol %) used as ligand. ^l^ Configuration determined based on the optical rotation, by comparison with literature.

**Table 2:**
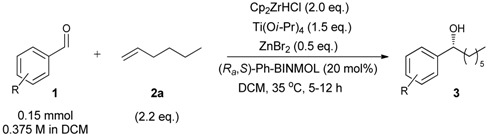
Enantioselective catalysed addition of 1-hexene (**2a**) to aromatic aldehydes—Scope of the reaction ^a^.

Entry	Product	Conv. (%) ^b^	Undesired Arylmethanol (%) ^b^	Yield (%) ^c^	*ee* (%) ^d^
1	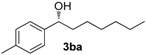	94	6	74	91 (*R*)
2	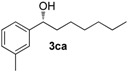	77	15 ^e^	54	89 (*R*)
3	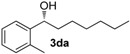	54	28 ^f^	49	76 (*R*)
4	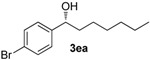	87	10	56	91 (*R*)
5 ^g^	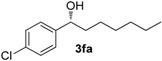	92	6	59	90 (*R*)
6	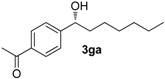	76	4 ^h^	32	94 (*R*)
7 ^f^	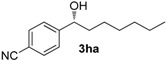	81	19	58	87 (*R*)
8	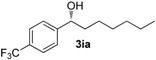	69	28	55	87 ^i^ (*R*)

^a^ Reaction conditions: **3** (0.15 mmol, 1.0 eq.), (*R*_a_,*S*)-Ph-BINMOL (0.2 eq.), Ti(O*^i^*Pr)_4_ (1.5 eq.), 1-hexene (2.2 eq.), Cp_2_ZrHCl (2.0 eq.), ZnBr_2_ (0.5 eq.), DCM (0.375 M), 35 °C, 5–12 h. ^b^ Determined by GC-MS. ^c^ Isolated yield after flash chromatography. ^d^ Determined by Chiral GC. Configuration based on literature data (see supplementary material for details). ^e^ 8% of dehydration product **4** was observed by GC-MS. ^f^ 18% of dehydration product **4** was observed by GC-MS. ^g^ The reaction was carried out in DCM (0.3 M). ^h^ 19% of **5** was observed by GC-MS. ^i^ Determined on the corresponding acetate derivative (see supplementary material for further details).

**Table 3:** Enantioselective catalysed addition of alkenes to benzaldehyde—Scope of the reaction ^a^.

Entry	Product	Conv. (%) ^b^	Yield (%) ^c^	*ee* (%) ^d^
1 ^e^	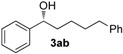	>99	93	77 (*R*)
2	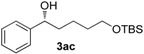	n.d.	42	88 (*R*)
3	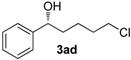	75 (10) ^f^	40	85 ^g^ (*R*)
4	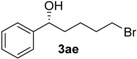	67	41	74 (*R*)
5	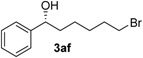	61	31	81 (*R*)

^a^ Reaction conditions: **1a** (0.15 mmol, 1.0 eq.), (*R*_a_,*S*)-Ph-BINMOL (0.2 eq.), Ti(O*^i^*Pr)_4_ (1.5 eq.), **2** (2.2 eq.), Cp_2_ZrHCl (2.0 eq.), ZnBr_2_ (0.5 eq.), DCM (0.375 M), 35 °C, 5–12 h. ^b^ Determined by GC-MS. ^c^ Isolated yield after flash chromatography. ^d^ Determined by Chiral GC. Configuration based on literature data (see supplementary material for details). ^e^ Reaction carried out with 3.0 eq. of **2** and 2.8 eq. of Cp_2_ZrHCl. ^f^ 15% of dehydration product **4** was observed by GC-MS. ^g^ Determined on the corresponding cyclised derivative **6** (see supplementary material for details).

## Supplementary Materials

Experimental methods and spectroscopic data for new compounds are available online.
