# Supplementary material for: Catalytic Enantioselective Addition of Organozirconium Reagents to Aldehydes
Source: Molecules. 2018 Apr 20;23(4):961. doi: 10.3390/molecules23040961 (PMC6017622; doi:10.3390/molecules23040961)

Supporting Information

Catalytic Asymmetric Addition of Organozirconium Reagents to Aldehydes

R. Solà,[a] M. Veguillas,[a] M. J. González-Soria,[a] N. Carter[a] M. A. Fernández-Ibáñez[,b] and B. Maciá[*,a]*

[a] Division of Chemistry & Environmental Science, Manchester Metropolitan University, Oxford Road, M1 5GD, Manchester, UK, E-mail: b.macia-ruiz@mmu.ac.uk

[b] Van 't Hoff Institute for Molecular Sciences. Science Park 904, 1090 GS, Amsterdam. E-mail: M.A.FernandezIbanez@uva.nl

Table of contents

General Considerations S-1

Experimental procedures S-2

GC and HPLC data S-8

NMR spectra for new compounds S-20

IR spectra for new compounds S-23

General Considerations: ^{1}H NMR and ^{13}C NMR have been recorded on a JEOL® ECS-400 (400 and 100.6 MHz, respectively) using $CDCl_3$ as solvent. Chemical shift values are reported in ppm with TMS as internal standard ($CDCl_3$: δ 7.26 for ^{1}H-NMR, δ 77.0 for ^{13}C-NMR). Data are reported as follows: chemical shifts, multiplicity (s= singlet, quint= quintuplet, m= multiplet), coupling constants (Hz), and integration. IR spectra were recorded on Nicolet® 380 FT/IR – Fourier Transform Infrared Spectrometer. Only the most significant frequencies have been considered during the characterization, and have been reported in cm^{-1}. High resolution mass spectra have been measured on an Agilent Technologies® 6540 Ultra-High-Definition (UHD) Accurate-Mass equipped with a time of flight (Q-TOF) analyzer and the samples were ionized by ESI techniques and introduced through a high pressure liquid chromatography (HPLC) model Agilent Technologies® 1260 Infinity Quaternary LC system. The GC chromatograms (for both conversion and enantioselectivity determination) have been recorded using an Agilent Technologies® 7890A GC System and a Hewlett Packard® 5890 Series II GC System, with a CycloSil-β (Agilent Technologies, 30 m × 0.25 mm) and a CP-Chiralsil-DEX CB (Varian, 25 m ×

0.25 mm) column, respectively; injector and detector temperatures: 250 °C. HPLC analysis (for enantioselectivity determination) was carried out on a *Agilent 1100 Series* HPLC equipped with a G1315B diode array detector and a Quat Pump G1311A, using the columns Lux 5μ Cellulose-1 (Phenomenex®, 250 mm × 4.60 mm) and Lux 5μ Cellulose-3 (Phenomenex®, 250 mm × 4.60 mm). Optical rotations were measured on a Bellingham + Stanley® ADP 440+ Polarimeter with a 0.5 cm cell (*c* given in g/100 mL).

All reactions were monitored by thin-layer chromatography using precoated sheets of silica gel 60, 0.25 mm thick (F254 Merck KGaA®). The components were visualized by UV light (254 nm) and phosphomolybdic acid or $KMnO_4$ staining. Flash column chromatography was done using Geduran® Silica gel 60, 40-63 microns RE. The eluent used is mentioned in each particular case. All glassware employed during inert atmosphere experiments was flame-dried under a stream of dry argon. All liquid aldehydes were freshly distilled before use. Alkenes reagents were purchased from Sigma-Aldrich or Fisher and used without further purification. Anhydrous THF, DCM, toluene and Et_2O were obtained from a Pure Solv™ Solvent Purification Systems.

Ligand (R_a,*S*)-Ph-BINMOL was prepared according to literature procedures[1] from (*R*)-BINOL, purchased from Manchester Organics.

Racemic alcohols **3aa-3ia** were synthesised from the addition of hexylmagnesium bromide to the corresponding aldehyde. Racemic **3ab** was synthesised using the general procedure below for the addition of the corresponding alkenes to benzaldehyde, using racemic BINOL as ligand. Racemic **3ac,**[2,3] **3ae**[2] and **3af**[4] were prepared according to literature procedures.

EXPERIMENTAL PROCEDURES

General procedure for the catalytic enantioselective 1,2-addition of alkenes to aldehydes: To a stirred suspension of Cp_2ZrHCl (77 mg, 0.30 mmol, 2.0 eq.) in dry DCM (0.3 mL) at RT, the corresponding alkene (0.33 mmol, 2.2 eq.) was added dropwise and the solution was stirred at RT for

---

[1] Fernández-Mateos, E.; Maciá, B.; Yus, M., *Adv. Syn. Cat.* **2013**, *355*, 1249–1254.

[2] Ketone precursor (5-bromo-1-phenylpentan-1-one) was prepared as by: (a) Wagner, P. J.; Lindstrom, M. J.; Sedon, J. H.; Ward, D. R., *J. Am. Chem. Soc.* **1981**, *103*, 3842. Reduction of the ketone precursor was carried out accordingly to: (b) Yamakawa, T.; Kinoshita, H.; Miura, K., *J. Organomet. Chem.* **2013**, *724*, 129, to get racemic 5-bromo-1-phenylpentan-1-ol (**3ae**).

[3] Racemic **3ae** was converted into 1-phenylpentane-1,5-diol as by: (a) Tranmer, G. K.; Keech, P.; Tam, W*., Chem. Commun*. **2000**, 863–864, and subsequently protected: (b) Brown, R. T.; Mayalarp, S. P.; Watts, J., *J. Chem. Soc., Perkin Trans. 1* **1997**, 1633–1638.

[4] Ketone precursor (6-bromo-1-phenylhexan-1-one) was prepared as by: Cavallaro, R. A. ; Filocamo, L.; Galuppi, A.; Galione, A.; Brufani, M.; Genazzani, A. A., *J. Med. Chem.* **1999**, *42*, 2527. Reduction of 6-bromo-1-phenylhexan-1-one was carried out according to reference 2b.

30 min. The mixture turned into a clear yellow solution, which indicated the successful formation of the organozirconium reagent. Next, flamed dried $ZnBr_2$ (0.08 mmol, 0.5 eq.) was added into the solution and the mixture was stirred at RT for 2 min. Subsequently, a solution of $Ti(O^iPr)_4$ (0.23 mmol, 1.5 eq.) and (R_a,*S*)-Ph-BINMOL (20 mol%) in dry DCM (0.1 mL) was added and stirred for further 2 min at RT. Finally, the aldehyde (0.15 mmol) was added and the solution was stirred at 35 °C for 3-18 h (reaction was monitored by TLC). [Note that liquid aldehydes were previously distilled before its addition whilts solid aldehydes were dissolved in dry DCM (0.1 or 0.2 mL depending on its solubility) and added to the solution]. The reaction was quenched by the addition of water (1 mL). The layers were separated, and the aqueous layer was extracted with Et_2O (3 × 10 mL). The combined organic layers were dried with anhydrous $MgSO_4$, filtered and concentrated under vacuum. The crude reaction product was purified by flash silica gel chromatography.

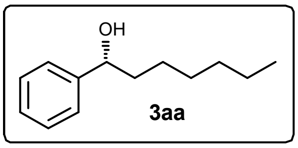

(*R*)-1-phenylheptanol (3aa):[5] Obtained as a colourless oil after purification by column chromatography (Hex/EtOAc 95:5). **Yield:** 87%. ***ee*:** 93%. $[\alpha]_D^{25}$ = +16.7 (*c* 8.4, $CHCl_3$). [lit.[212] $[\alpha]_D^{25}$ = +31.8 (*c* 1.1, $CHCl_3$) for 99% *ee*]. **^{1}H NMR** (400 MHz, $CDCl_3$) δ 7.46–7.24 (m, 5H), 4.68–4.55 (m, 1H), 2.76 (s, 1H), 1.95–1.65 (m, 2H), 1.48–1.25 (m, 8H), 0.95 (t, *J* = 7.1 Hz, 3H). **^{13}C NMR** (100.6 MHz, $CDCl_3$) δ 145.1, 128.4, 127.4, 126.0, 74.6, 39.2, 31.9, 29.3, 25.8, 22.7, 14.1. *ee* determination by chiral **GC** analysis, Cyclosil β column, T = 150 °C, P = 15.9 psi, retention times: t_r(*S*) = 28.7 min, t_r(*R*) = 30.2 min (major enantiomer).

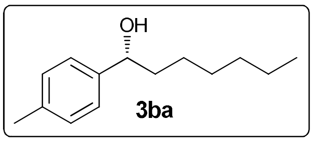

(*R*)-1-p-tolylheptan-1-ol (3ba):[6] Obtained as a white solid after purification by column chromatography (Hex/EtOAc 95:5). **Yield:** 74%. ***ee*:** 91%. **M_p** = 34–37 °C. $[\alpha]_D^{25}$ = +18.7 (*c* 7.5, $CHCl_3$). [lit.[210] $[\alpha]_D^{26}$ = +27.7 (*c* 1.1, $CHCl_3$) for 89% *ee*]. **IR** (ATR) 3344, 2924, 2855, 1456, 1041, 816. **^{1}H NMR** (400 MHz, $CDCl_3$) δ 7.25–7.10 (m, 4H), 4.65–4.58 (m, 1H), 2.34 (s, 3H), 1.80–1.60 (m, 2H), 1.45–1.28 (m, 8H), 0.86 (t, *J* = 7.6 Hz, 3H). **^{13}C NMR** (100.6 MHz, $CDCl_3$) δ 142.0, 137.1, 129.1, 125.8, 74.5, 39.0, 31.7, 29.2, 25.8, 22.6, 21.1, 14.1. *ee* determination by chiral **GC** analysis, CP Chirasil-DEX CB column, T = 140 °C, P = 6 psi, retention times: t_r(*S*) = 75.0 min, t_r(*R*) = 76.9 min (major enantiomer).

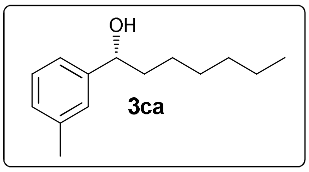

(*R*)-1-m-tolylheptan-1-ol (3ca): Obtained as a yellowish oil after purification by column chromatography (Hex/EtOAc 95:5). **Yield:** 34%. ***ee*:** 89%. $[\alpha]_D^{25}$ = +19.3 (*c* 5.7, $CHCl_3$). **IR** (ATR) 3348, 2925, 2856, 1457, 784, 702. **^{1}H NMR** (400 MHz, $CDCl_3$) δ 7.30–7.05 (m, 4H), 4.65–4.57 (m, 1H),

---

[5] (a) Salvi, N. A.; Chattopadhyay, S., *Tetrahedron* **2001,** *57*, 2833–2839. (b) Zong, H.; Huang, H. Y.; Song, L., *Tetrahedron-Asymmetry* **2016,** *27*, 1069–1074.
[6] Kumar, R.; Kawasaki, H.; Harada, T., *Org. Lett.* **2013,** *15*, 4198–4201.

2.35 (s, 3H), 1.85 (s broad, 1H), 1.83–1.60 (m, 2H), 1.45–1.20 (m, 8H), 0.87 (t, J = 6.8 Hz, 3H). **^{13}C NMR** (100.6 MHz, $CDCl_3$) δ 145.1, 138.2, 128.4, 128.3, 126.7, 123.1, 74.9, 39.2, 31.9, 29.3, 26.0, 22.7, 21.6, 14.2. **HRMS** (+ESI): m/z calculated for $C_{14}H_{22}ONa$ $[M+Na]^+$: 229.1563. Found: 229.1562. *ee* determination by chiral **GC** analysis, CP Chirasil-DEX CB column, T = 140 °C, P = 6 psi, retention times: $t_r(S)$ = 79.8 min, $t_r(R)$ = 82.3 min (major enantiomer).

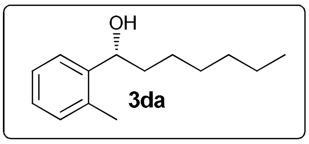

(*R*)-1-o-tolylheptan-1-ol (3da): [7] Obtained as a yellowish oil after purification by column chromatography (Hex/EtOAc 95:5). **Yield:** 49%. ***ee*:** 76%. **$[\alpha]_D^{25}$** = +28.6 (*c* 4.9, $CHCl_3$). **IR** (ATR) 3347, 2925, 2855, 1459, 1043, 754. **^{1}H NMR** (400 MHz, $CDCl_3$) δ 7.50–7.10 (m, 4H), 4.95–4.88 (m, 1H), 2.33 (s, 3H), 1.78 (s broad, 1H), 1.75–1.60 (m, 2H), 1.55–1.22 (m, 8H), 0.87 (t, J = 6.8 Hz, 3H). **^{13}C NMR** (100.6 MHz, $CDCl_3$) δ 143.2, 134.6, 130.5, 127.2, 126.4, 125.2, 70.9, 38.3, 31.9, 29.4, 26.2, 22.8, 19.2, 14.2. *ee* determination by chiral **GC** analysis, CP Chirasil-DEX CB column, T = 140 °C, P = 6 psi, retention times: $t_r(S)$ = 77.5 min, $t_r(R)$ = 83.8 min (major enantiomer).

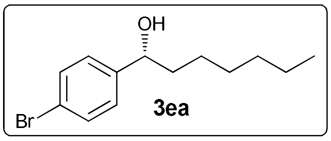

(*R*)-1-(4-bromophenyl)heptan-1-ol (3ea):[7] Obtained as a white solid after purification by column chromatography (Hex/EtOAc 95:5). **Yield:** 56%. ***ee*:** 91%. **M_p** = 35–37 °C. **$[\alpha]_D^{25}$** = +18.6 (*c* 7.5, $CHCl_3$). [lit.[8] **$[\alpha]_D^{25}$** = +23.3 (*c* 0.6, $CHCl_3$) for 99% *ee*]. **IR** (ATR) 3299, 2920, 2851, 1483, 1404, 1007. **^{1}H NMR** (400 MHz, $CDCl_3$) δ 7.50–7.16 (m, 4H), 4.66–4.58 (m, 1H), 1.91 (s broad, 1H), 1.82–1.58 (m, 2H), 1.45–1.15 (m, 8H), 0.87 (t, J = 7.2 Hz, 3H). **^{13}C NMR** (100.6 MHz, $CDCl_3$) δ 144.0, 131.6, 127.8, 121.3, 74.2, 39.2, 31.9, 29.3, 25.8, 22.7, 14.2. *ee* determination by chiral **HPLC** analysis, Phenomenex® Lux Cellulose-1, Hex/*i*-PrOH 95:5 flow = 1 mL/min, retention times: $t_r(S)$ = 8.4 min, $t_r(R)$ = 8.9 min (major enantiomer).

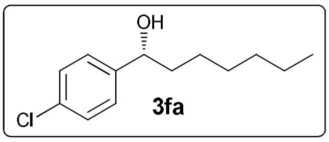

(*R*)-1-(4-chlorophenyl)heptan-1-ol (3fa):6 Obtained as a white solid after purification by column chromatography (Hex/EtOAc 95:5 to 90:10). **Yield:** 59%. ***ee*:** 90%. **M_p** = 33–35 °C. **$[\alpha]_D^{25}$** = +18.1 (*c* 6.6, $CHCl_3$). [lit.8 **$[\alpha]_D^{25}$** = +26.1 (*c* 0.3, $CHCl_3$) for 99% *ee*]. **IR** (ATR) 3280, 2923, 2854, 1466, 1089, 827. **^{1}H NMR** (400 MHz, $CDCl_3$) δ 7.35–7.22 (m, 4H), 4.68–4.60 (m, 1H), 1.89 (s broad, 1H), 1.82–1.58 (m, 2H), 1.44–1.18 (m, 8H), 0.87 (t, J = 6.8 Hz, 3H). **^{13}C NMR** (100.6 MHz, $CDCl_3$) δ 143.5, 133.2, 128.7, 127.4, 74.1, 39.3, 31.9, 29.3, 25.8, 22.7, 14.2. *ee* determination by chiral **HPLC** analysis, Phenomenex® Lux Cellulose-1, Hex/*i*-PrOH 95:5 flow = 1 mL/min, retention times: $t_r(S)$ = 7.6 min, $t_r(R)$ = 8.0 min (major enantiomer).

---

[7] Kabalka, G. W.; Wu, Z. Z.; Ju, Y. H., *Tetrahedron* **2001,** *57*, 1663–1670.
[8] Cho, J.; Lee, J.; Park, J.; Kim, M. J., *Tetrahedron-Asymmetry* **2015,** *26*, 840–845.

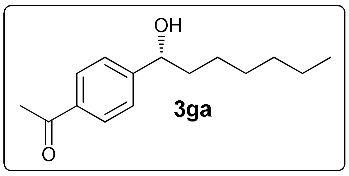
3ga

(*R*)-1-[4-(1-oxidanylheptyl)phenyl]ethanone (3ga): Obtained as a white solid after purification by column chromatography (Hex/EtOAc 80:20). **Yield:** 32%. ***ee*:** 94%. **M_p** = 37–39 °C. **$[\alpha]_D^{25}$** = +15.8 (*c* 3.8, $CHCl_3$). **IR** (ATR) 3283, 2925, 2854, 1678, 1606, 1266. **^{1}H NMR** (400 MHz, $CDCl_3$) δ 7.94 (d, *J* = 8.4 Hz, 2H), 7.44 (d, *J* = 8.4 Hz, 2H), 4.78–4.70 (m, 1H), 2.60 (s, 3H), 1.98 (s broad, 1H), 1.85–1.64 (m, 2H), 1.46–1.18 (m, 8H), 0.87 (t, *J* = 7.2 Hz, 3H). **^{13}C NMR** (100.6 MHz, $CDCl_3$) δ 198.0, 150.4, 136.5, 128.7, 126.1, 74.3, 39.4, 31.9, 29.3, 26.8, 25.7, 22.7, 14.2. **HRMS** (+ESI): m/z calculated for $C_{15}H_{23}O_2$ $[M+H]^+$: 235.1693. Found: 235.1693. *ee* determination by chiral **HPLC** analysis, Phenomenex® Lux Cellulose-1, Hex/*i*-PrOH 95:5 flow = 1 mL/min, retention times: t_r(*R*) = 17.8 min (major enantiomer), t_r(*S*) = 19.1 min.

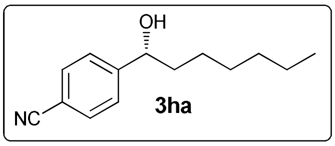
3ha

(*R*)-4-(hydroxyheptyl)-benzonitrile (3ha):[9] Obtained as a colourless oil after purification by column chromatography (Hex/EtOAc 95:5 to 80:20). **Yield:** 58%. ***ee*:** 87%. **$[\alpha]_D^{25}$** = +17.5 (*c* 6.3, $CHCl_3$). **IR** (ATR) 3433, 2927, 2856, 2228, 1609, 839, 732. **^{1}H NMR** (400 MHz, $CDCl_3$) δ 7.63 (d, *J* = 8.4 Hz, 2H), 7.46 (d, *J* = 8.4 Hz, 2H), 4.78–4.70 (m, 1H), 2.07 (s broad, 1H), 1.82–1.58 (m, 2H), 1.46–1.18 (m, 8H), 0.87 (t, *J* = 6.8 Hz, 3H). **^{13}C NMR** (100.6 MHz, $CDCl_3$) δ 150.3, 132.3, 126.5, 118.9, 111.1, 73.8, 39.3, 31.7, 29.1, 25.5, 25.6, 14.0. *ee* determination by chiral **HPLC** analysis, Phenomenex® Lux Cellulose-1, Hex/*i*-PrOH 95:5 flow = 0.5 mL/min, retention times: t_r(*S*) = 29.4 min, t_r(*S*) = 30.7 min (major enantiomer).

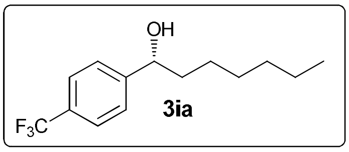
3ia

(*R*)-1-[4-(trifluoromethyl)phenyl]heptan-1-ol (3ia):[10] Obtained as a yellowish oil after purification by column chromatography (Hex/EtOAc 95:5). **Conversion:** 69%. ***ee*:** 87%. **$[\alpha]_D^{25}$** = +0.14 (*c* 1.0, $CHCl_3$). *ee* was determined by chiral **HPLC** analysis on derivative **3ia'**. **IR** (ATR) 3336, 2929, 2858, 1620, 1323, 1122. **^{1}H NMR** (400 MHz, $CDCl_3$) δ 7.60 (d, *J* = 8.0 Hz, 2H), 7.46 (d, *J* = 8.0 Hz, 2H), 4.78–4.70 (m, 1H), 1.95 (s broad, 1H), 1.84–1.64 (m, 2H), 1.48–1.20 (m, 8H), 0.87 (t, *J* = 6.8 Hz, 3H). **^{13}C NMR** (100.6 MHz, $CDCl_3$) δ 148.9, 129.6 (q, *J* = 128.8 Hz), 126.1, 125.4 (q, *J* = 14.8 Hz), 122.8, 74.0, 39.3, 31.7, 29.1, 25.6, 22.6, 14.1.

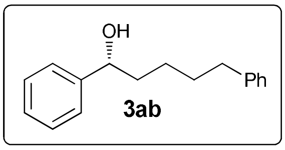
3ab

(*R*)-1,5-diphenyl-pentan-1-ol (3ab):[11] Obtained as a colourless oil after purification by column chromatography (Hex/EtOAc 90:10). **Yield:** 93%. ***ee*:** 77%. **$[\alpha]_D^{25}$** = +5.4 (*c* 11.2, $CHCl_3$). **IR** (ATR) 3381, 2931, 2856, 1494, 1452, 696. **^{1}H NMR** (400 MHz, $CDCl_3$) δ 7.38–7.13 (m, 10H), 4.72–4.62 (m, 1H),

[9] Keh, C. C. K.; Wei, C. M.; Li, C. J., *J. Am. Chem. Soc.* **2003,** *125*, 4062–4063.
[10] Hamada, S.; Furuta, T.; Wada, Y.; Kawabata, T., *Angew. Chem. Int. Ed.* **2013,** *52*, 8093–8097.
[11] Endo, K.; Ohkubo, T.; Hirokami, M.; Shibata, T., *J. Am. Chem. Soc.* **2010,** *132*, 11033–11035.

2.59 (t, *J* = 8.0 Hz, 2H), 1.89–1.60 (m, 4H), 1.53–1.43 (m, 1H), 1.40–1.25 (m, 1H). **^{13}C NMR** (100.6 MHz, $CDCl_3$) δ 144.8, 142.6, 128.5, 128.4, 128.3, 127.5, 125.9, 125.6, 74.6, 38.9, 35.8, 31.4, 25.5. *ee* determination by chiral **GC** analysis, Cyclosil β column, T = 180 °C, P = 15.9 psi, retention times: t_r(*S*) = 20.0 min, t_r(*R*) = 20.6 min (major enantiomer).

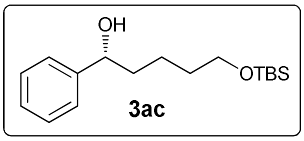

(*R*)-5-(*tert*-butyl-dimethyl-silanyloxy)-1-phenyl-pentan-1-ol (3ac): [12] Obtained as a yellowish oil after purification by column chromatography (Hex/EtOAc 95:5). **Yield:** 42%. ***ee*:** 88%. **$[\alpha]_D^{25}$** = +13.3 (*c* 6.0, $CHCl_3$). **IR** (ATR) 3376, 2927, 2856, 1253, 1096, 833. **^{1}H NMR** (400 MHz, $CDCl_3$) δ 7.36–7.22 (m, 5H), 4.70–4.64 (m, 1H), 3.59 (t, *J* = 6.4 Hz, 2H), 1.95 (s broad, 1H), 1.88–1.26 (m, 6H), 0.87 (s, 9H), 0.03 (s, 6H). **^{13}C NMR** (100.6 MHz, $CDCl_3$) δ 145.0, 128.6, 127.6, 126.0, 74.8, 63.2, 39.0, 32.7, 29.8, 26.1, 22.3, 18.5, –5.1. *ee* determination by chiral **HPLC** analysis, Phenomenex® Lux Cellulose-1, Hex/*i*-PrOH 95:5 flow = 0.5 mL/min, retention times: t_r(*R*) = 13.3 min (major enantiomer), t_r(*S*) = 15.0 min.

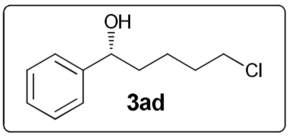

(*R*)-5-chloro-1-phenylpentanol (3ad):[13] Obtained as a colourless oil after purification by column chromatography (Hex/EtOAc 95:5 to 90:10). **Conversion:** 75%. ***ee*:** 85%. **$[\alpha]_D^{25}$** = +0.19 (*c* 1.0, $CHCl_3$). *ee* was determined by chiral **HPLC** analysis on derivative **6**. **IR** (ATR) 3355, 2918, 2863, 1453, 1027, 699. **^{1}H NMR** (400 MHz, $CDCl_3$) δ 7.40–7.24 (m, 5H), 4.68 (dd, *J* = 5.6, 7.6 Hz, 1H), 3.52 (t, *J* = 6.4 Hz, 2H), 1.89 (s broad, 1H), 1.88–1.36 (m, 6H). **^{13}C NMR** (100.6 MHz, $CDCl_3$) δ 144.6, 128.5, 127.7, 125.8, 74.4, 44.9, 38.2, 32.5, 23.2.

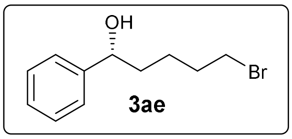

(R)-5-bromo-1-phenylpentan-1-ol (3ae):[14] Obtained as a pale yellow oil after purification by column chromatography (Hex/EtOAc 95:5). **Conversion:** 67%. ***ee*:** 74%. **$[\alpha]_D^{25}$** = +0.04 (*c* 1.2, $CHCl_3$). **IR** (ATR) 3366, 2934, 2862, 1452, 1245, 1055, 1026, 759, 699.**^{1}H NMR** (400 MHz, $CDCl_3$) δ 7.37–7.25 (m, 5H), 4.66 (dd, *J* = 5.6, 7.6 Hz, 1H), 3.38 (t, *J* = 6.8 Hz, 2H), 1.91–1.41 (m, 7H). **^{13}C NMR** (100.6 MHz, $CDCl_3$) δ 144.6, 128.6, 127.8, 125.9, 74.5, 38.2, 33.7, 32.7, 24.6. *ee* determination by chiral **GC** analysis, CycloSil-β column, T = 140 °C, P = 6 psi, retention times: t_r(*S*) = 123.7 min, t_r(*R*) = 127.1 min (major enantiomer).

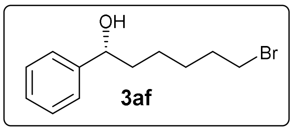

(R)-6-bromo-1-phenylhexan-1-ol (3af):[15] Obtained as a yellow oil after purification by column chromatography (Hex/EtOAc 95:5 to 80:20).

[12] (a) Zlotorzynska, M.; Zhai, H. M.; Sammis, G. M., *J. Org. Chem.* **2010,** *75*, 864–872. (b) Zhai, H. M.; Wickenden, J. G.; Sammis, G. M., *Synlett* **2010**, 3035–3038.
[13] Foubelo, F.; Abou, A.; Yus, M., *Eur. J. Org. Chem.* **2005**, 5089–5093.
[14] Kumar, R.; Kawasaki, H.; Harada, T., *Org. Lett.* **2013**, 15, 4198-4201.
[15]Miura, K.; Tomita, M.; Yamada, Y.; Hosomi, A., *J. Org. Chem.* **2007**, 72, 787-792.

Conversion: 61%. ***ee***: 82%. **[α]**$_D^{25}$ = +0.12 (*c* 1.4, $CHCl_3$). **IR** (ATR) 3223, 2932, 2857, 1452, 1056, 1026, 755, 699. **^{1}H NMR** (400 MHz, $CDCl_3$) δ 7.30–7.18 (m, 5H), 4.59 (t, *J* = 6.6 Hz, 1H), 3.31 (t, *J* = 6.8 Hz, 2H), 1.81-1.58 (m, 5H), 1.42–1.32 (m, 1H), 1.26-1.17 (m, 1H). **^{13}C NMR** (100.6 MHz, $CDCl_3$) δ 144.8, 128.6, 127.8, 126.0, 74.6, 38.9, 34.0, 32.8, 28.2, 25.1. *ee* determination by chiral **GC** analysis, CP Chirasil-DEX CB column, T = 140 °C, P = 6 psi, retention times: t_r(*S*) = 95.1 min, t_r(*R*) = 96.3 min (major enantiomer).

General procedure for the synthesis of acetates derivatives: In a flame dried Schlenk tube, the aliphatic alcohol **3ia** (0.2 mmol) was dissolved in anhydrous DCM (2 mL, 0.1 M) at 0 °C and Et_3N (56 µL, 0.4 mmol, 2 eq.), DMAP (2.6 mg, 0.02 mmol, 0.1 eq.) and acetic anhydride (44 µL, 0.4 mmol, 2 eq.) were added sequentially. The reaction mixture was stirred at RT for 12 h. The reaction was quenched with water (2 mL), extracted with Et_2O (3 × 5 mL) and the combined organic layers were dried over $MgSO_4$ and concentrated under vacuum. The crude product was purified by chromatographic column to provide **3ia’**.

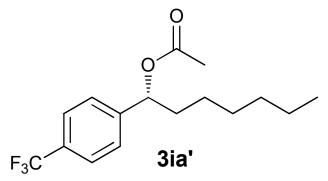

3ia'

(*R*)-1-[4-(trifluoromethyl)phenyl]heptan-1-yl acetate (3ia’): Obtained as a yellowish oil after purification by column chromatography (Hex/EtOAc 98:2). **Yield**: 55%. ***ee***: 87%. **[α]**$_D^{25}$ = +29.5 (*c* 8.1, $CHCl_3$). **IR** (ATR) 2930, 2859, 1739, 1622, 1323, 1123. **^{1}H NMR** (400 MHz, $CDCl_3$) δ 7.60 (d, *J* = 8.0 Hz, 2H), 7.43 (d, *J* = 8.0 Hz, 2H), 5.78–5.70 (m, 1H), 2.08 (s, 3H), 1.98–1.66 (m, 2H), 1.40–1.16 (m, 8H), 0.87 (t, *J* = 7.2 Hz, 3H). **^{13}C NMR** (100.6 MHz, $CDCl_3$) δ 170.3, 144.9, 130.0 (q, *J* = 129.2 Hz), 126.7, 125.4 (q, *J* = 14.8 Hz), 122.7 (q, *J* = 280.0 Hz), 75.5, 36.3, 31.6, 28.9, 25.3, 22.5, 21.2, 14.0. **HRMS** (+ESI): m/z calculated for $C_{16}H_{25}NO_2F_3$ $[M+NH_4]^+$: 320.1839. Found: 320.1837. *ee* determination by chiral **HPLC** analysis, Phenomenex® Lux Cellulose-1, Hexane 100 flow = 0.5 mL/min, retention times: t_r(*R*) = 23.1 min (major enantiomer), t_r(*S*) = 24.5 min.

General procedure for the synthesis of 2-substituted chiral tetrahydropyrans: In a flame dried Schlenk tube, the corresponding chiral 4-halogenbutyl alcohol (**3ad** or **3ae**) (0.15 mmol) was dissolved in anhydrous THF (1.5 mL). Then, KO^{t}Bu (50 mg, 0.45 mmol, 3 eq.) was added to the previous solution and the resulting suspension was stirred at RT for 18 h. The reaction was quenched with water (2 mL) and the crude was extracted with EtOAc (3 × 5 mL). The combined organic layers were dried over $MgSO_4$ and concentrated under vacuum. The crude product was purified by chromatographic column to provide **6**.

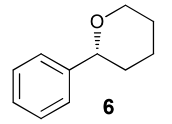

6

(*R*)-2-Phenyltetrahydro-2*H*-pyran (6):[16] Obtained as a yellowish oil after purification by column chromatography (Hex/EtOAc 95:5). **Yield**: 84%. ***ee*:** 85%. **$[\alpha]_D$**^{25} = +21.4 (*c* 1.4, $CHCl_3$). **IR** (ATR) 2934, 2844, 1604, 1451, 1087, 697. **^{1}H NMR** (400 MHz, $CDCl_3$) δ 7.36–7.22 (m, 5H), 4.32 (dd, *J* = 11.2, 2.2 Hz, 1H), 4.18–4.11 (m, 1H), 3.62 (td, *J* = 11.6, 2.4 Hz, 1H), 1.98–1.89 (m, 1H), 1.87–1.78 (m, 1H), 1.76–1.52 (m, 4H). **^{13}C NMR** (100.6 MHz, $CDCl_3$) δ 143.3, 128.2, 127.2, 125.8, 80.1, 69.0, 34.0, 25.9, 24.0. *ee* determination by chiral **HPLC** analysis, Phenomenex® Lux Cellulose-3, Hex/^{i}PrOH 95:5 flow = 0.5 mL/min, retention times: t_r(*S*) = 16.4 min, t_r(*R*) = 17.7 min (major enantiomer).

[16] (a) Zhu, Q. L.; Gentry, E. C.; Knowles, R. R., *Ang. Chem. Int. Ed.* **2016,** *55*, 9969–9973. (b) Zhu, Q. L.; Gentry, E. C.; Knowles, R. R., *Angew. Chem.* **2016,** *128*, 10123–10127.

GC and HPLC data

(*R*)-1-phenylheptanol (3aa):

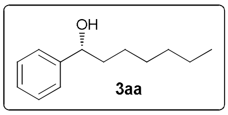

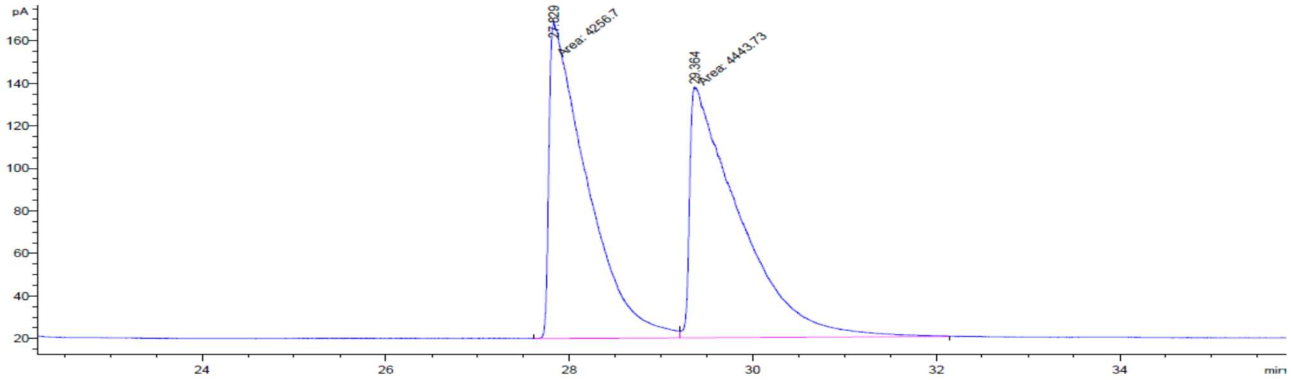

Peak #	RetTime [min]	Type	Width [min]	Area [pA*s]	Height [pA]	Area %
1	27.829	MF	0.4764	4256.70264	148.90456	48.92519
2	29.364	FM	0.6294	4443.72900	117.66441	51.07481
Totals :				8700.43164	266.56896	

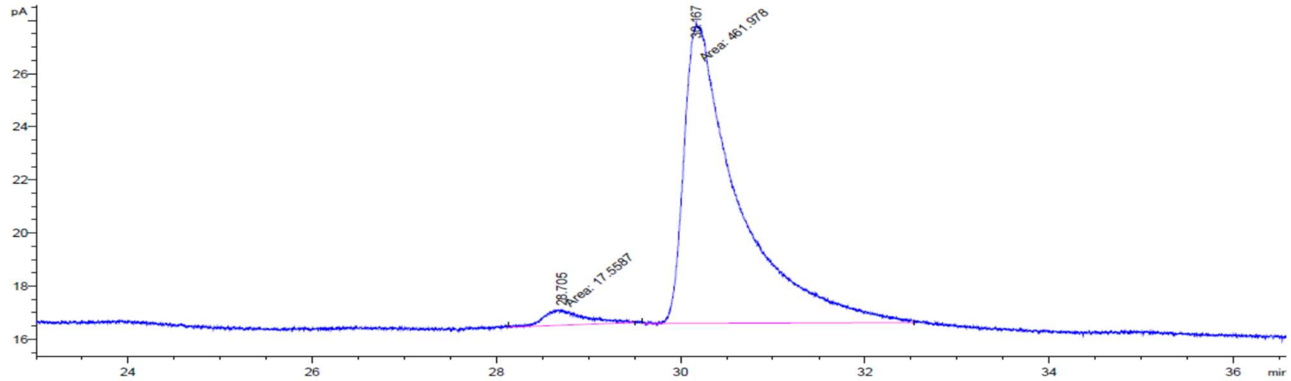

Peak #	RetTime [min]	Type	Width [min]	Area [pA*s]	Height [pA]	Area %
1	28.705	MM	0.4776	17.55868	6.12734e-1	3.66159
2	30.167	MM	0.6769	461.97800	11.37419	96.33841
Totals :				479.53667	11.98692	

(*R*)-1-p-tolylheptan-1-ol (3ba):

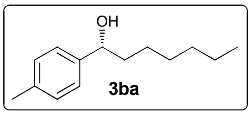

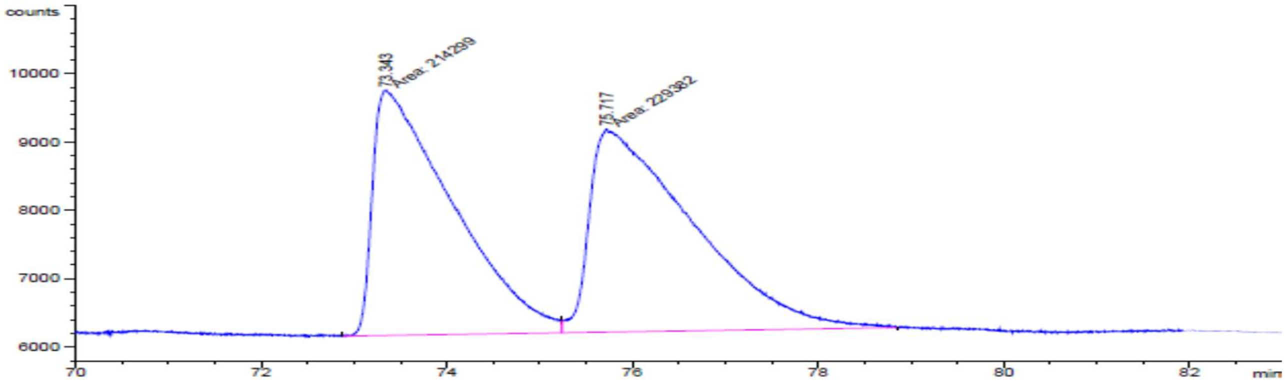

Peak #	RetTime [min]	Type	Width [min]	Area counts*s	Height [counts]	Area %
1	73.343	MF	0.9939	2.14299e5	3593.57153	48.30028
2	75.717	FM	1.2849	2.29382e5	2975.30518	51.69972

Totals : 4.43682e5 6568.87671

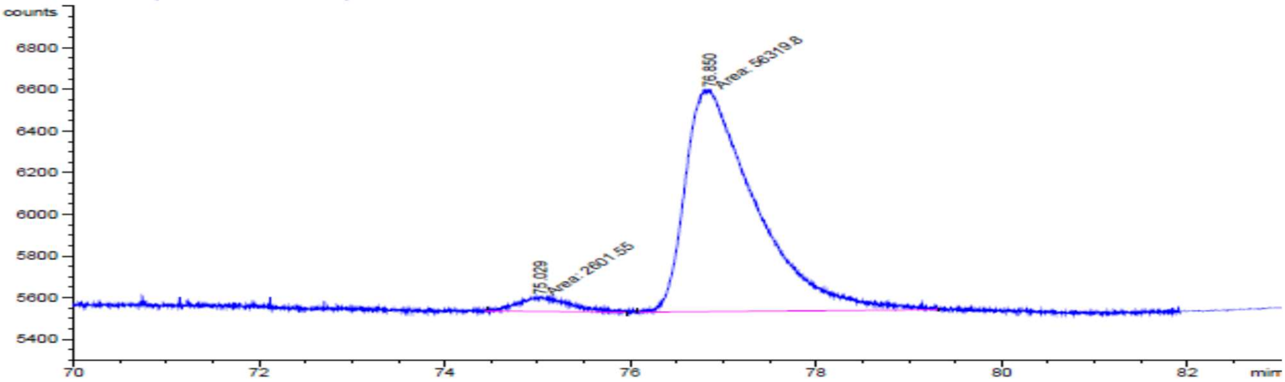

Peak #	RetTime [min]	Type	Width [min]	Area counts*s	Height [counts]	Area %
1	75.029	MM	0.6280	2601.55322	69.04525	4.41530
2	76.850	MM	0.8836	5.63198e4	1062.33398	95.58470

Totals : 5.89213e4 1131.37923

(*R*)-1-m-tolylheptan-1-ol (3ca):

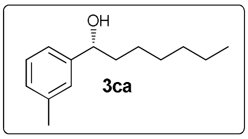

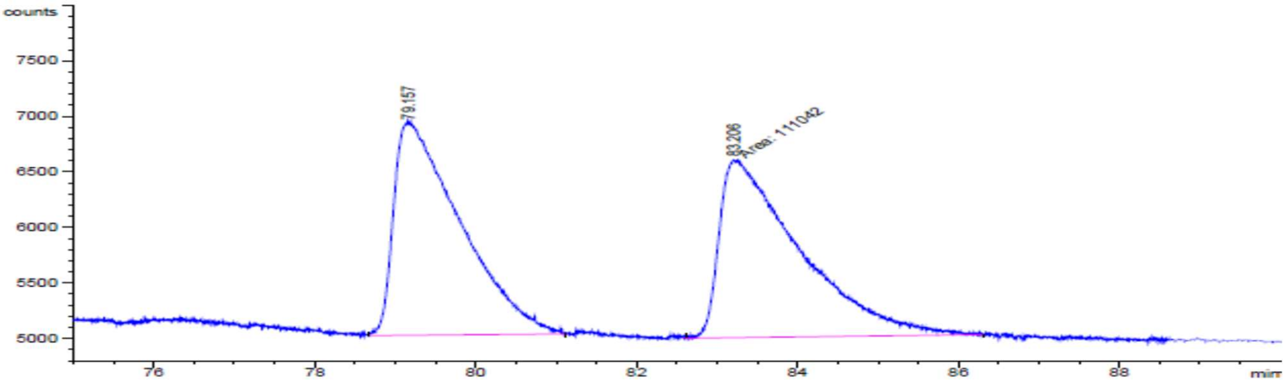

Peak #	RetTime [min]	Type	Width [min]	Area counts*s	Height [counts]	Area %
1	79.157	PB	0.7291	1.10009e5	1918.25977	49.76627
2	83.206	MM	1.1548	1.11042e5	1602.60181	50.23373
Totals :				2.21051e5	3520.86157	

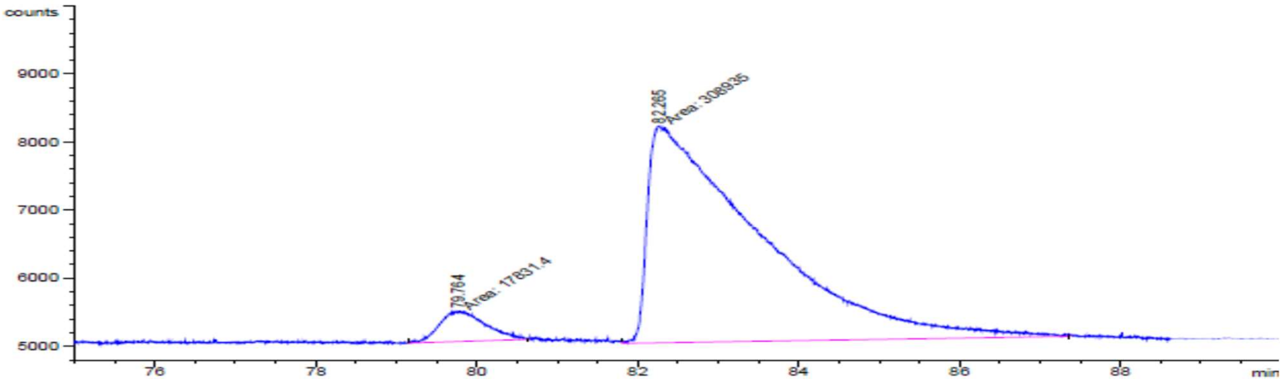

Peak #	RetTime [min]	Type	Width [min]	Area counts*s	Height [counts]	Area %
1	79.764	MM	0.6697	1.78314e4	443.74429	5.45693
2	82.265	MM	1.6169	3.08935e5	3184.42603	94.54307
Totals :				3.26766e5	3628.17032	

(*R*)-1-o-tolylheptan-1-ol (3da):

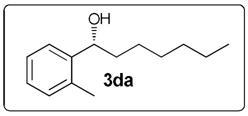

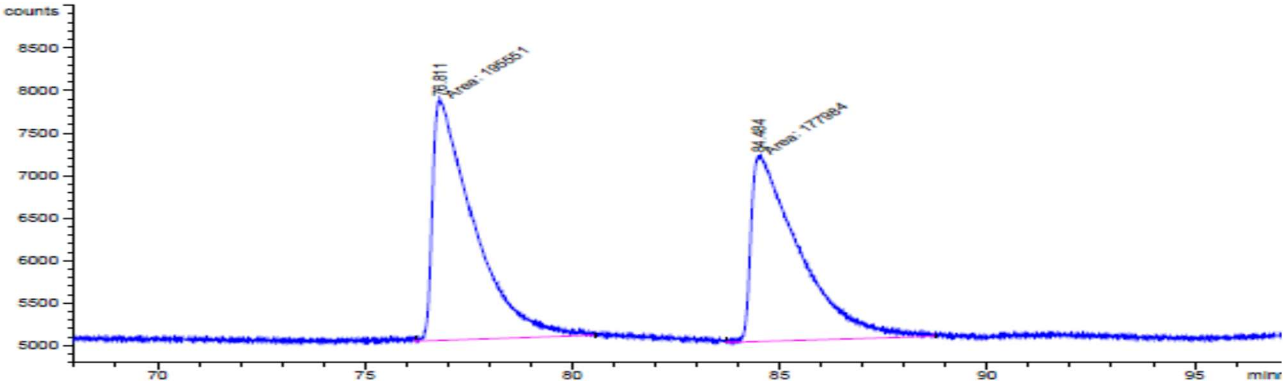

Peak #	RetTime [min]	Type	Width [min]	Area counts*s	Height [counts]	Area %
1	76.811	MM	1.1508	1.95551e5	2832.20483	52.35149
2	84.484	MM	1.3564	1.77984e5	2186.98950	47.64851

Totals : 3.73535e5 5019.19434

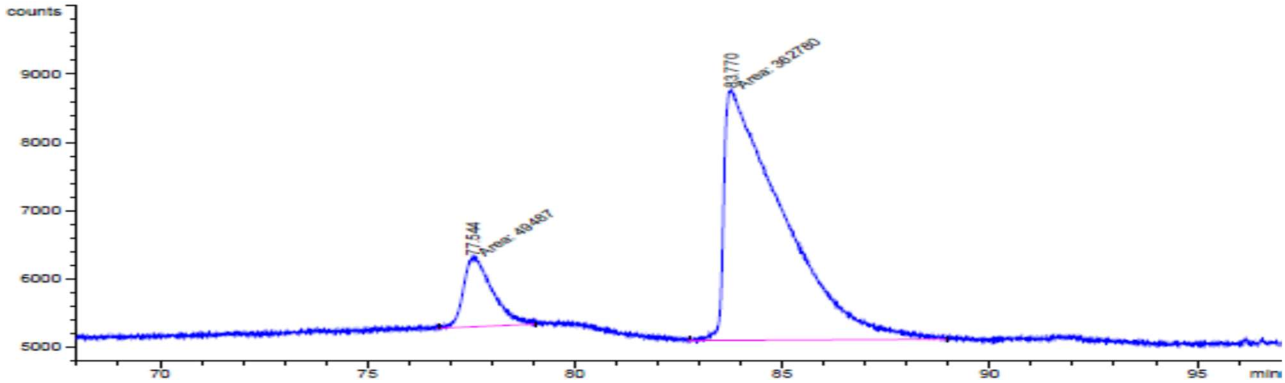

Peak #	RetTime [min]	Type	Width [min]	Area counts*s	Height [counts]	Area %
1	77.544	MM	0.8208	4.94870e4	1004.86932	12.00363
2	83.770	MM	1.6535	3.62780e5	3656.72119	87.99637

Totals : 4.12267e5 4661.59052

(*R*)-1-(4-bromophenyl)heptan-1-ol (3ea)

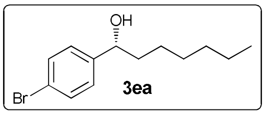

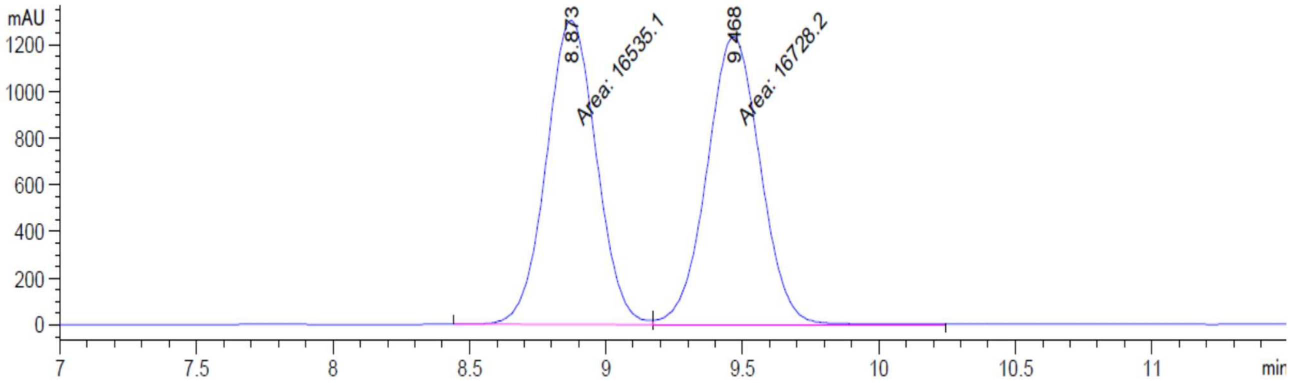

Peak #	RetTime [min]	Type	Width [min]	Area [mAU*s]	Height [mAU]	Area %
1	8.873	MF	0.2116	1.65351e4	1302.07886	49.7097
2	9.468	FM	0.2265	1.67282e4	1231.18530	50.2903
Totals :				3.32633e4	2533.26416	

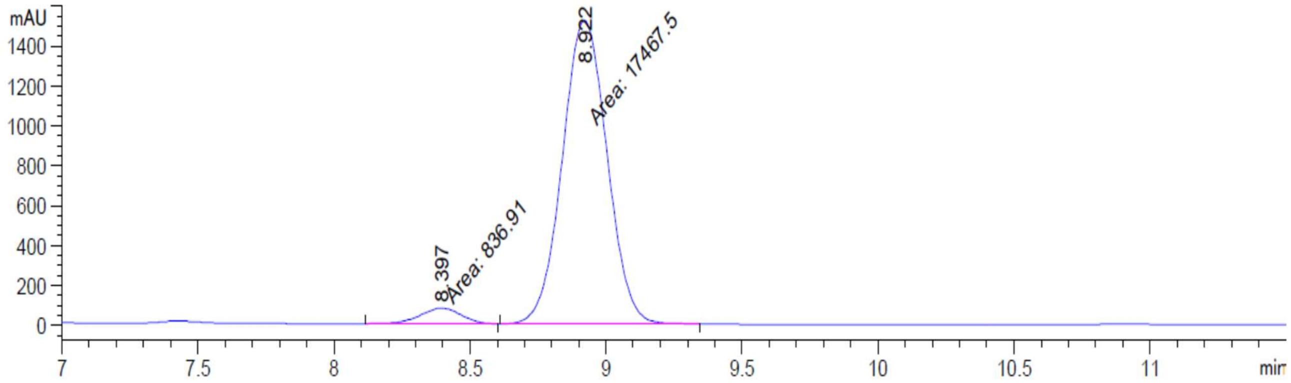

Peak #	RetTime [min]	Type	Width [min]	Area [mAU*s]	Height [mAU]	Area %
1	8.397	MM	0.1774	836.91034	78.64093	4.5722
2	8.922	MM	0.1910	1.74675e4	1524.39380	95.4278
Totals :				1.83044e4	1603.03473	

(*R*)-1-(4-chlorophenyl)heptan-1-ol (3fa):

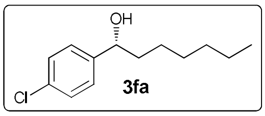

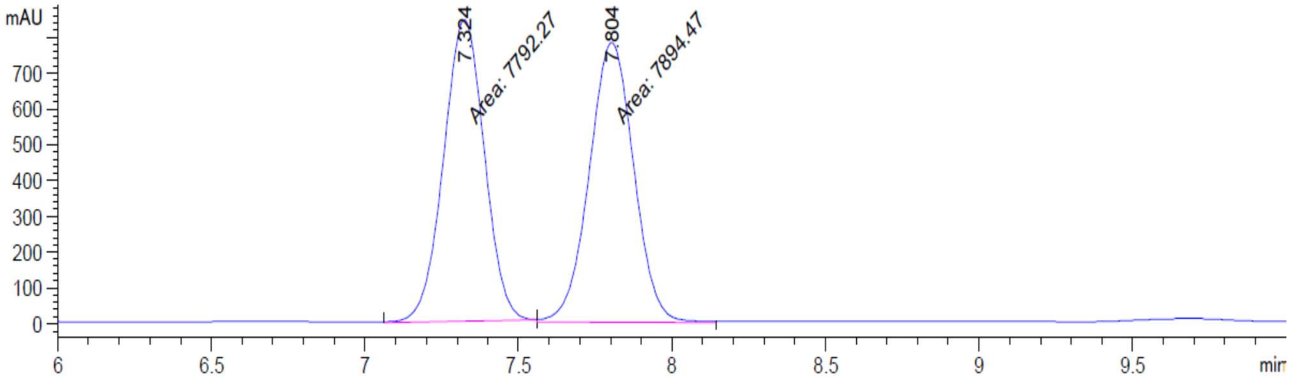

Peak #	RetTime [min]	Type	Width [min]	Area [mAU*s]	Height [mAU]	Area %
1	7.324	MM	0.1541	7792.26660	843.04407	49.6742
2	7.804	MM	0.1683	7894.46582	781.59479	50.3258

Totals : 1.56867e4 1624.63885

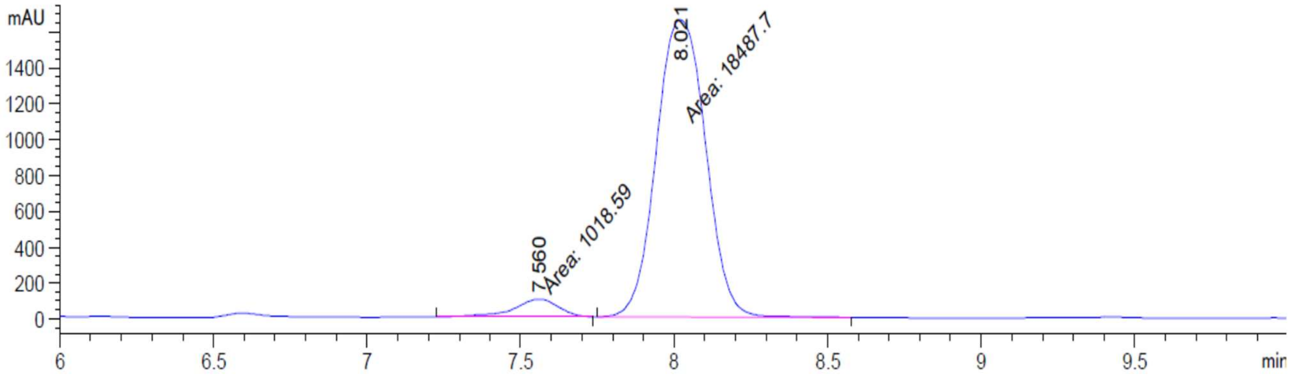

Peak #	RetTime [min]	Type	Width [min]	Area [mAU*s]	Height [mAU]	Area %
1	7.560	MM	0.1707	1018.59308	99.44547	5.2219
2	8.021	MM	0.1858	1.84877e4	1658.42297	94.7781

Totals : 1.95063e4 1757.86845

(*R*)-1-[4-(1-oxidanylheptyl)phenyl]ethanone (3ga):

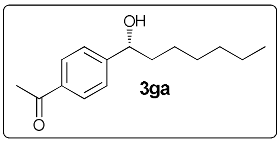

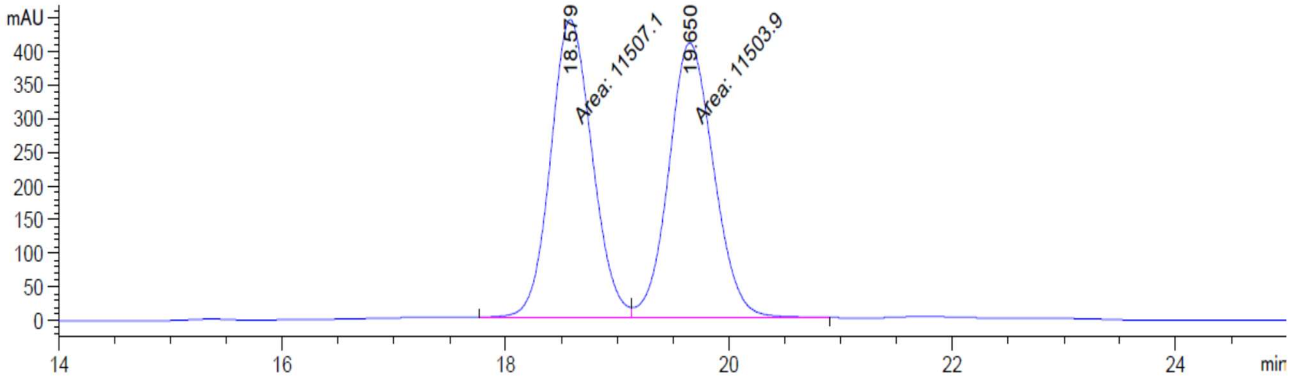

Peak #	RetTime [min]	Type	Width [min]	Area [mAU*s]	Height [mAU]	Area %
1	18.579	MF	0.4329	1.15071e4	443.04874	50.0069
2	19.650	FM	0.4694	1.15039e4	408.45468	49.9931

Totals : 2.30111e4 851.50342

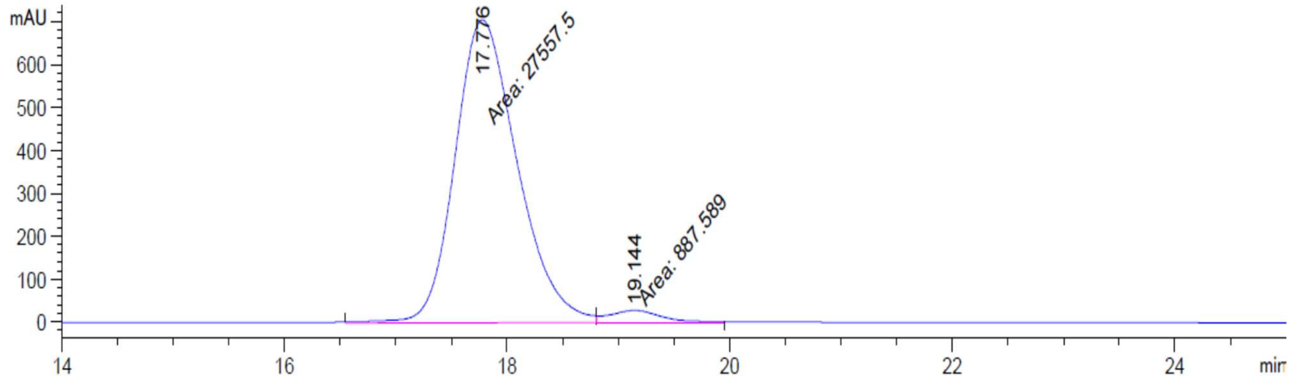

Peak #	RetTime [min]	Type	Width [min]	Area [mAU*s]	Height [mAU]	Area %
1	17.776	MF	0.6503	2.75575e4	706.30695	96.8796
2	19.144	FM	0.5319	887.58875	27.81071	3.1204

Totals : 2.84451e4 734.11765

(*R*)-4-(hydroxyheptyl)-benzonitrile (3ha):

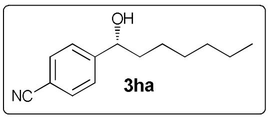

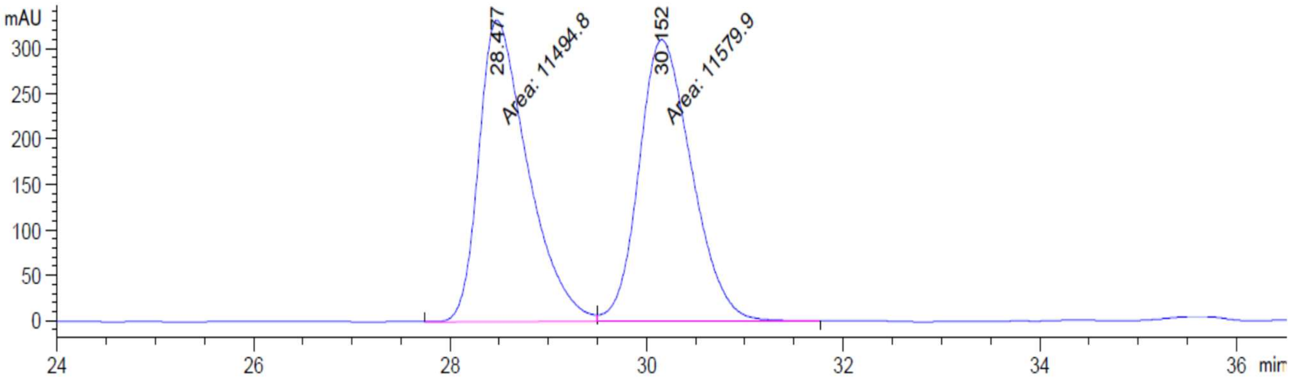

Peak #	RetTime [min]	Type	Width [min]	Area [mAU*s]	Height [mAU]	Area %
1	28.477	MF	0.5768	1.14948e4	332.16467	49.8155
2	30.152	FM	0.6221	1.15799e4	310.25717	50.1845

Totals : 2.30747e4 642.42184

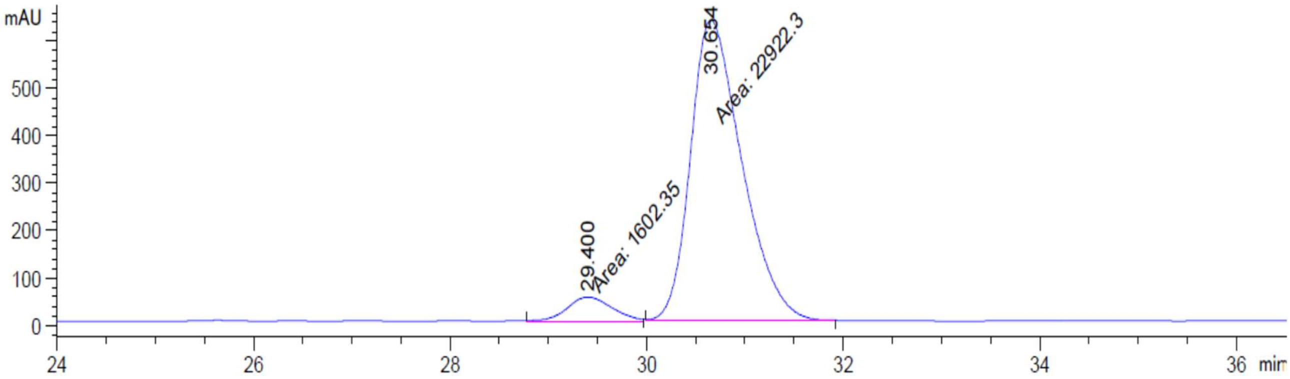

Peak #	RetTime [min]	Type	Width [min]	Area [mAU*s]	Height [mAU]	Area %
1	29.400	MM	0.5266	1602.34998	50.71410	6.5336
2	30.654	MM	0.6028	2.29223e4	633.76605	93.4664

Totals : 2.45246e4 684.48015

(*R*)-1,5-diphenyl-pentan-1-ol (3ab):

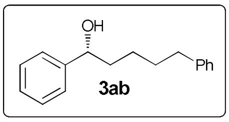

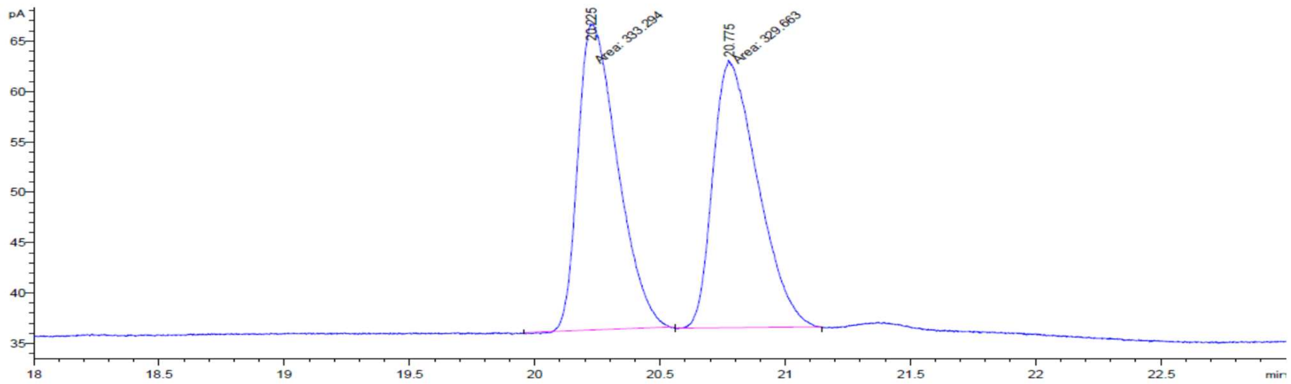

Peak #	RetTime [min]	Type	Width [min]	Area [pA*s]	Height [pA]	Area %
1	20.225	MM	0.1831	333.29446	30.34004	50.27390
2	20.775	MM	0.2069	329.66275	26.55744	49.72610
Totals :				662.95721	56.89748	

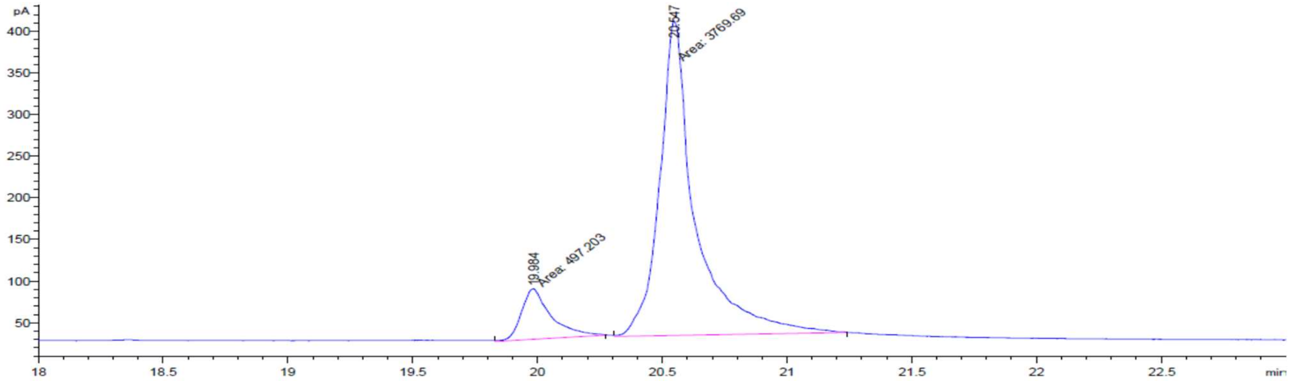

Peak #	RetTime [min]	Type	Width [min]	Area [pA*s]	Height [pA]	Area %
1	19.984	MM	0.1367	497.20337	60.60537	11.65259
2	20.547	MM	0.1665	3769.68774	377.26749	88.34741
Totals :				4266.89111	437.87286	

(*R*)-5-(*tert*-butyl-dimethyl-silanyloxy)-1-phenyl-pentan-1-ol (3ac):

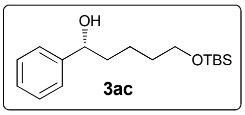

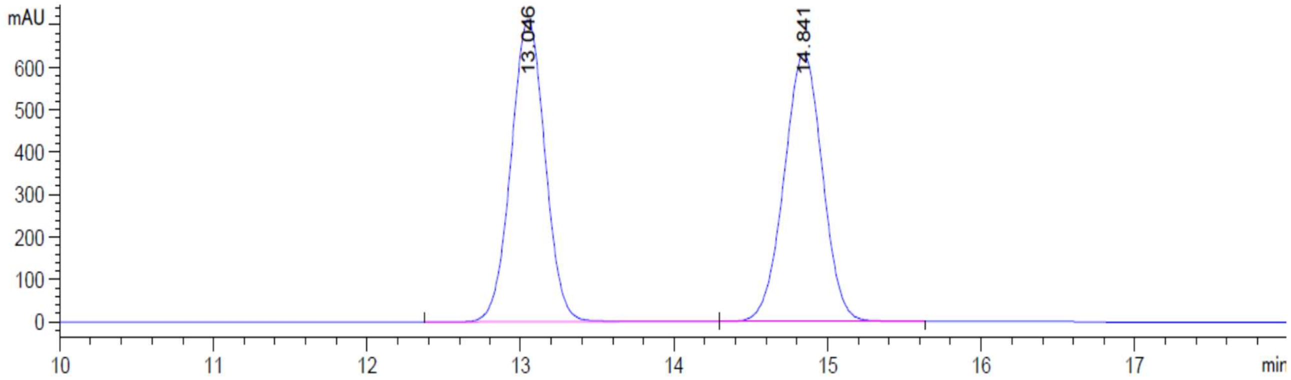

Peak #	RetTime [min]	Type	Width [min]	Area [mAU*s]	Height [mAU]	Area %
1	13.046	BB	0.2399	1.09671e4	711.15753	49.9977
2	14.841	BB	0.2710	1.09682e4	630.35175	50.0023
Totals :				2.19353e4	1341.50928	

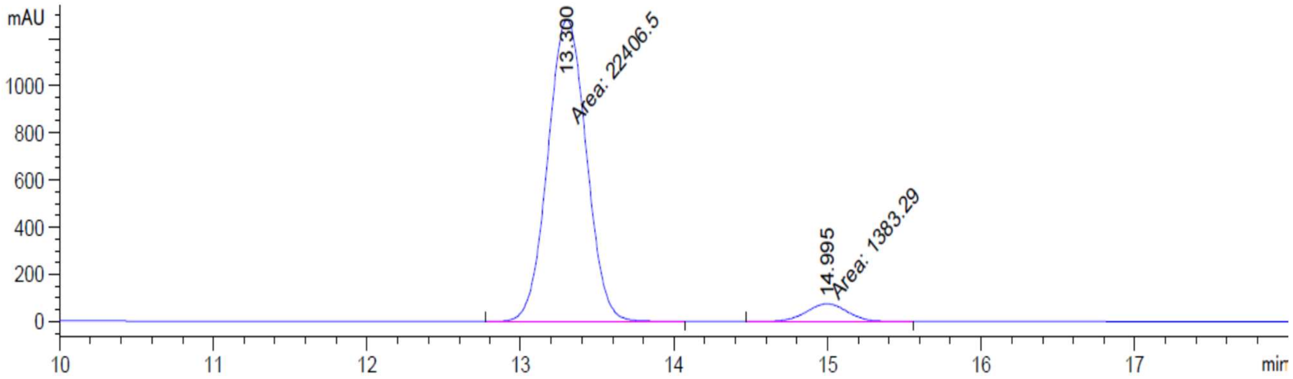

Peak #	RetTime [min]	Type	Width [min]	Area [mAU*s]	Height [mAU]	Area %
1	13.300	MM	0.2913	2.24065e4	1282.19336	94.1853
2	14.995	MM	0.3077	1383.29456	74.92508	5.8147
Totals :				2.37898e4	1357.11844	

(R)-5-bromo-1-phenylpentan-1-ol (3af):

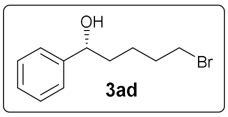

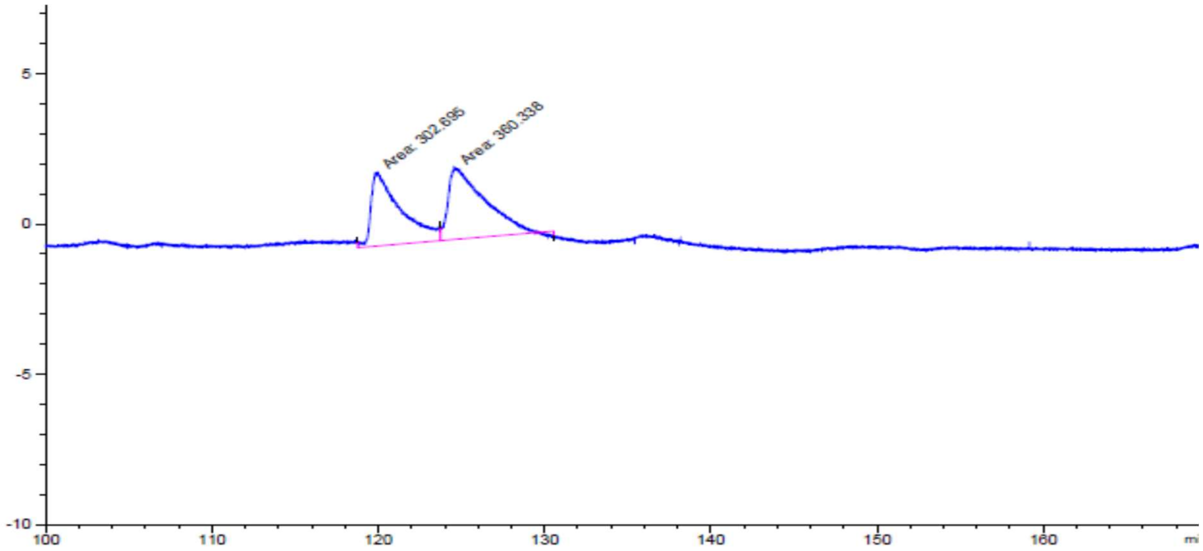

Peak #	RetTime [min]	Type	Width [min]	Area [pA*s]	Height [pA]	Area %
1	119.952	MF	2.0481	302.69467	2.46321	45.65304
2	124.661	FM	2.5016	360.33820	2.40068	54.34696
Totals :				663.03287	4.86388	

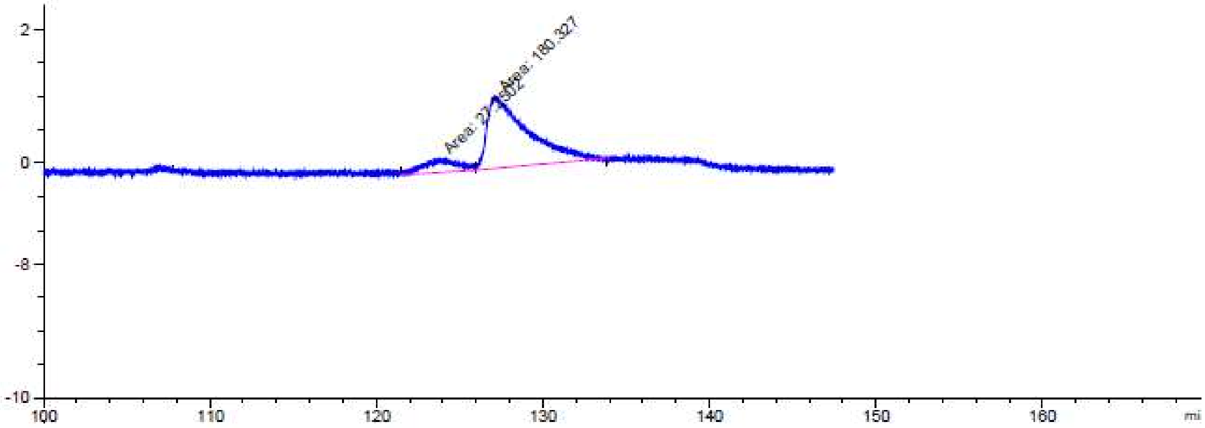

Peak #	RetTime [min]	Type	Width [min]	Area [pA*s]	Height [pA]	Area %
1	123.735	MM	1.9677	27.25018	2.30815e-1	13.12771
2	127.074	MM	2.7360	180.32742	1.09850	86.87229
Totals :				207.57760	1.32932	

(R)-6-bromo-1-phenylhexan-1-ol (3af):

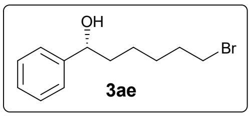

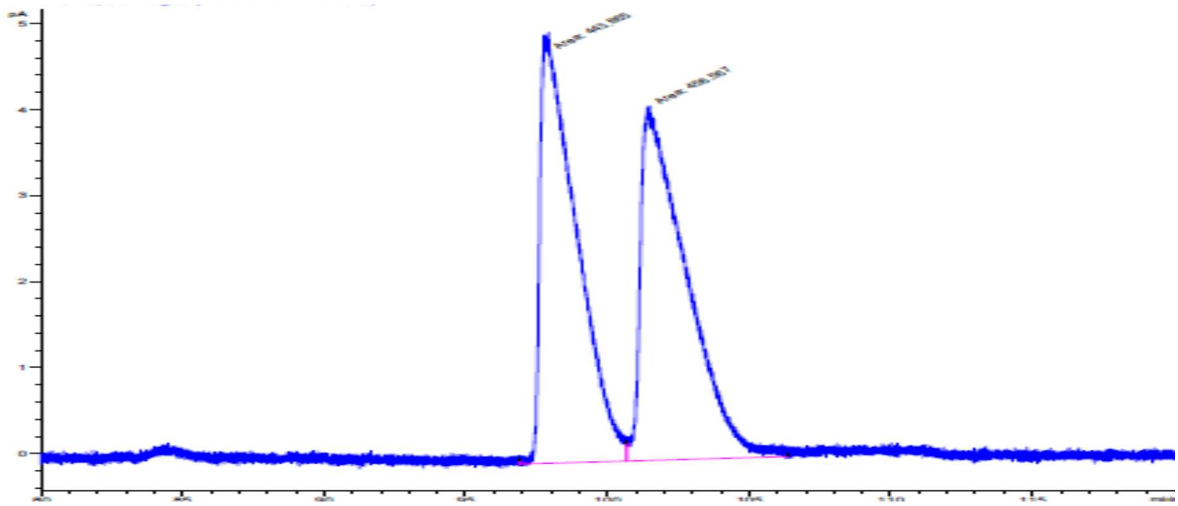

Peak #	RetTime [min]	Type	Width [min]	Area [pA*s]	Height [pA]	Area %
1	97.925	MF	1.4755	443.86526	5.01371	49.18545
2	101.506	FM	1.8501	458.56686	4.13090	50.81455
Totals :				902.43213	9.14461	

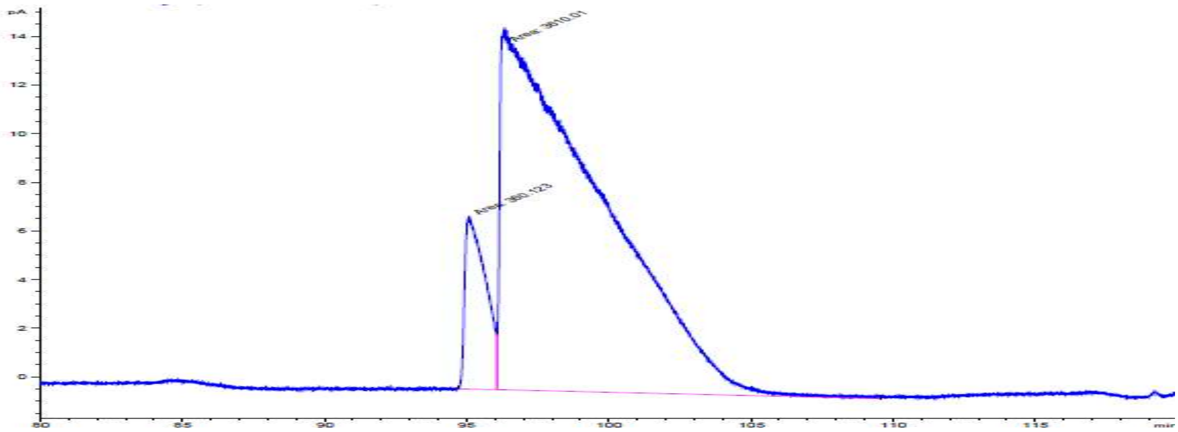

Peak #	RetTime [min]	Type	Width [min]	Area [pA*s]	Height [pA]	Area %
1	95.078	MF	0.8432	360.12320	7.11822	9.07080
2	96.341	FM	4.0338	3610.01245	14.91570	90.92920
Totals :				3970.13565	22.03392	

(*R*)-1-[4-(trifluoromethyl)phenyl]heptan-1-yl acetate (3ia'):

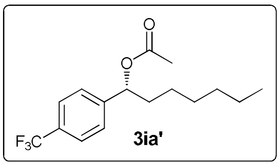

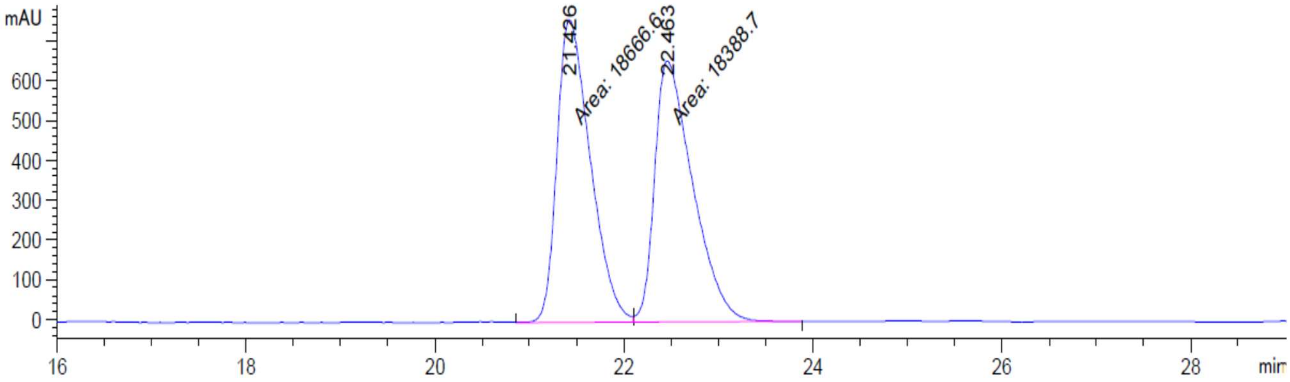

Peak #	RetTime [min]	Type	Width [min]	Area [mAU*s]	Height [mAU]	Area %
1	21.426	MF	0.4092	1.86666e4	760.34033	50.3750
2	22.463	FM	0.4671	1.83887e4	656.11487	49.6250
Totals :				3.70552e4	1416.45520	

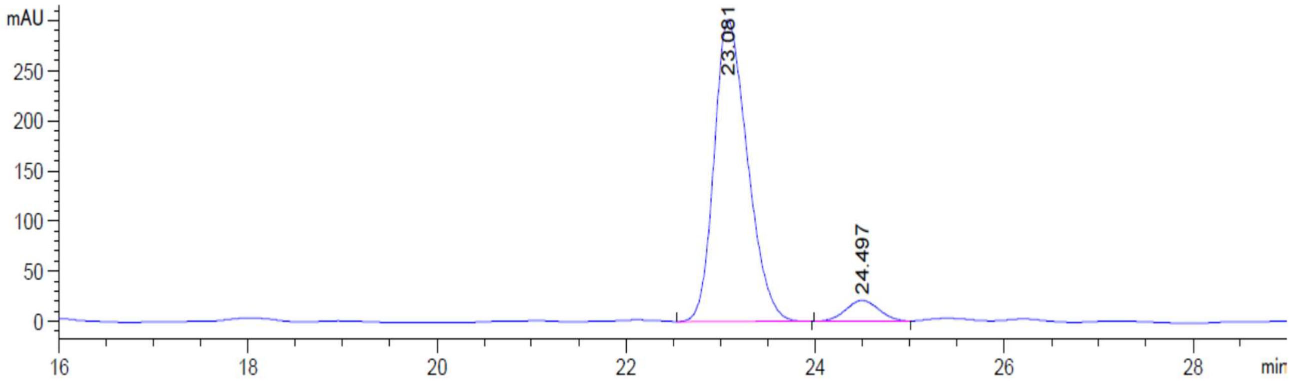

Peak #	RetTime [min]	Type	Width [min]	Area [mAU*s]	Height [mAU]	Area %
1	23.081	BB	0.3819	7376.80615	301.01224	93.7121
2	24.497	BV	0.3808	494.96994	20.56814	6.2879
Totals :				7871.77609	321.58037	

(*R*)-2-Phenyltetrahydro-2*H*-pyran (6)

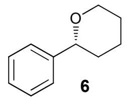

6

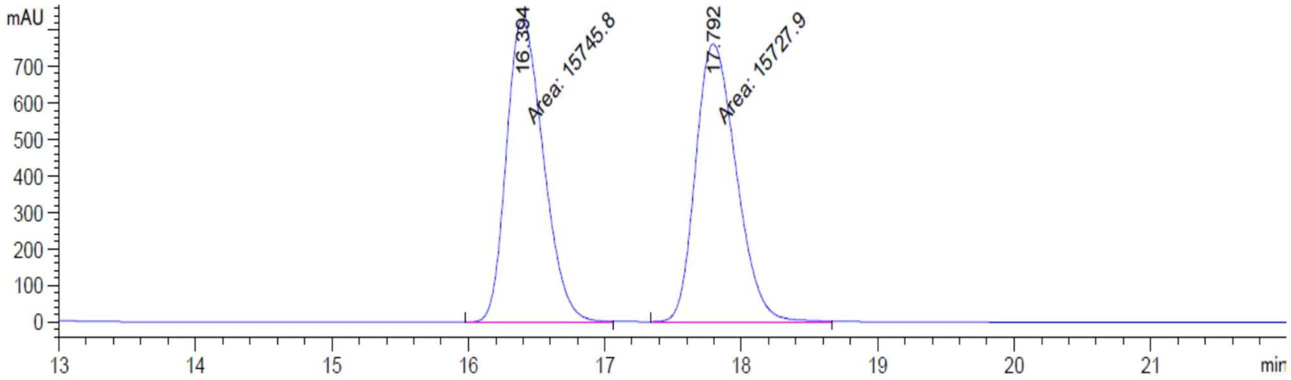

Peak #	RetTime [min]	Type	Width [min]	Area [mAU*s]	Height [mAU]	Area %
1	16.394	MM	0.3160	1.57458e4	830.39709	50.0285
2	17.792	MM	0.3444	1.57279e4	761.01373	49.9715
Totals :				3.14736e4	1591.41083	

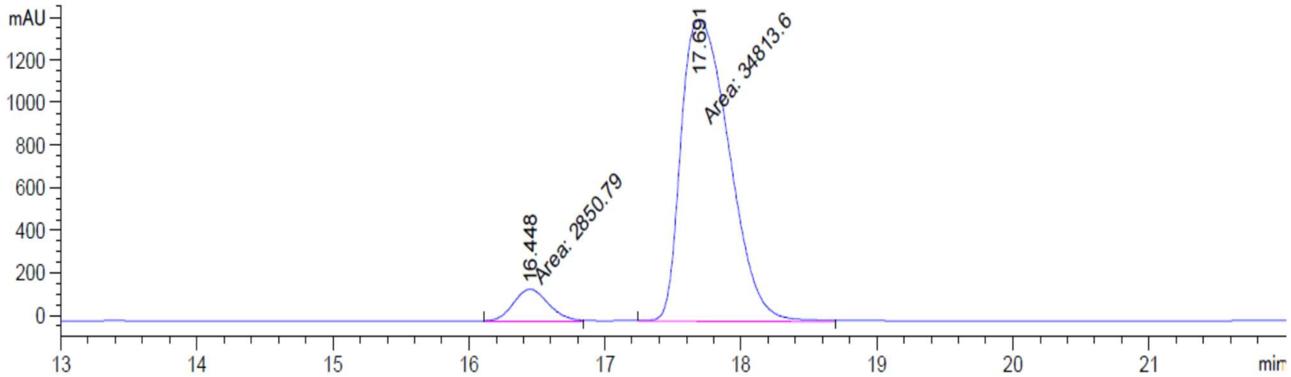

Peak #	RetTime [min]	Type	Width [min]	Area [mAU*s]	Height [mAU]	Area %
1	16.448	MM	0.3104	2850.79175	153.05565	7.5689
2	17.691	MM	0.4106	3.48136e4	1413.13550	92.4311
Totals :				3.76644e4	1566.19115	

NMR spectra for new compounds

(*R*)-1-m-tolylheptan-1-ol (3ca)

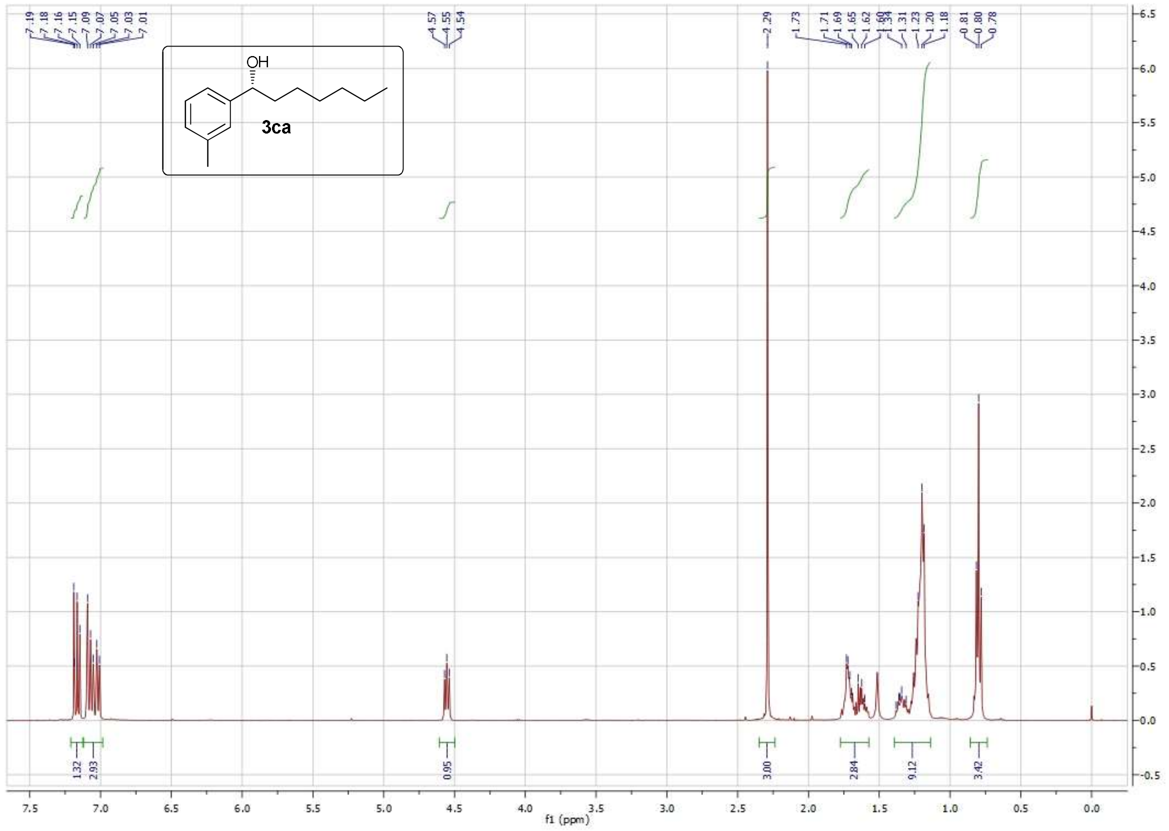

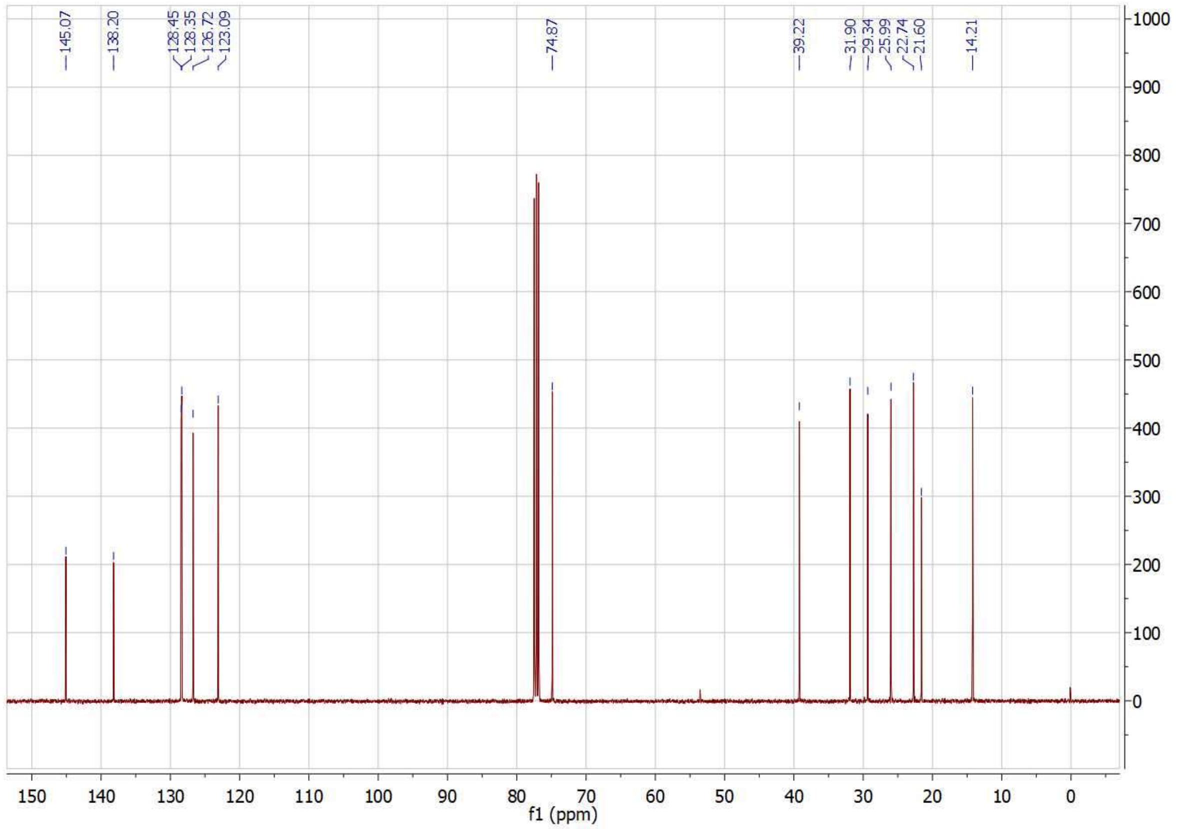

(*R*)-1-[4-(1-oxidanylheptyl)phenyl]ethanone (3ga)

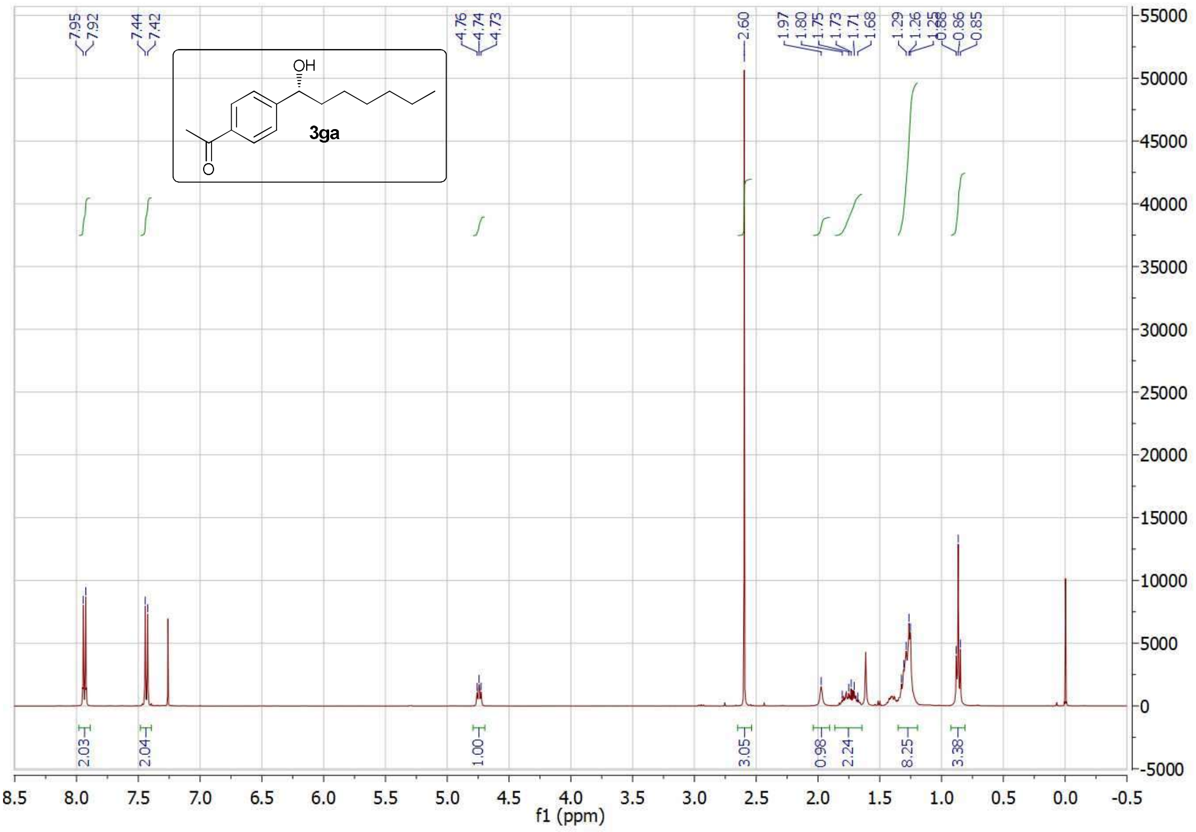

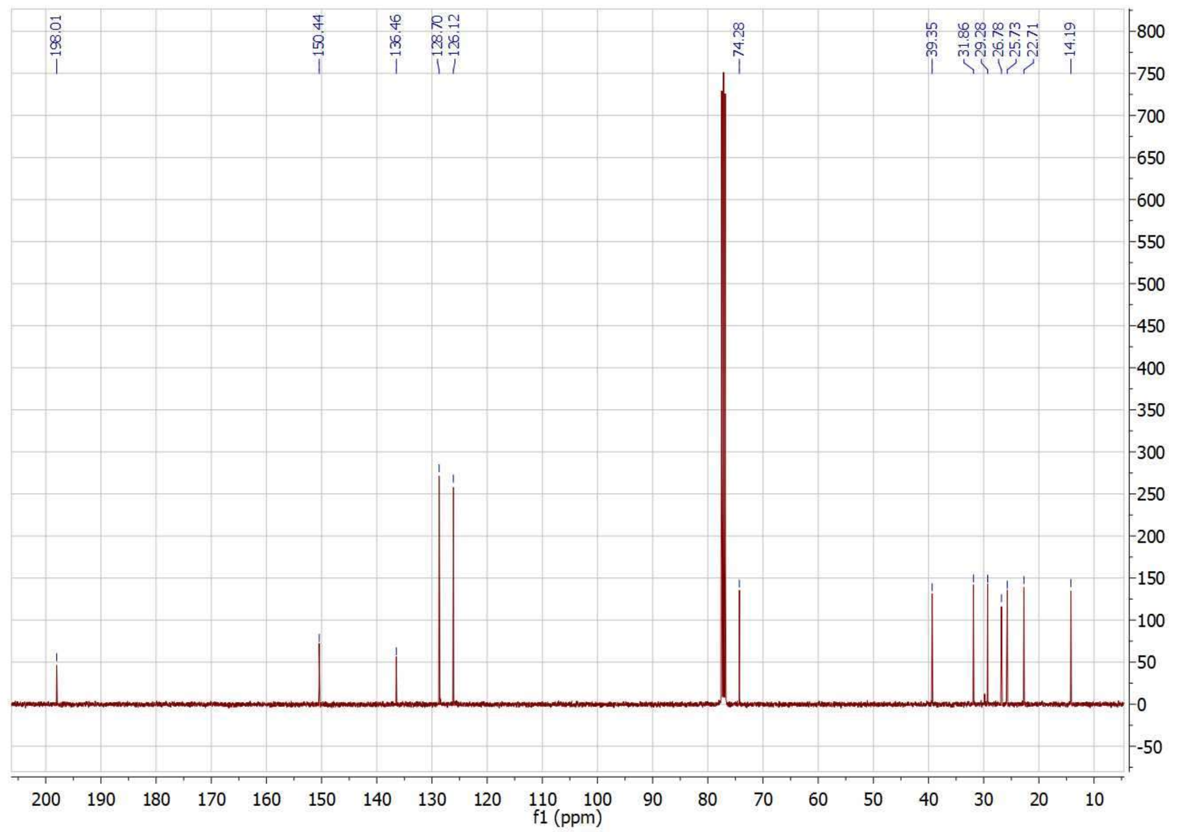

(*R*)-1-[4-(trifluoromethyl)phenyl]heptan-1-yl acetate (3ia')

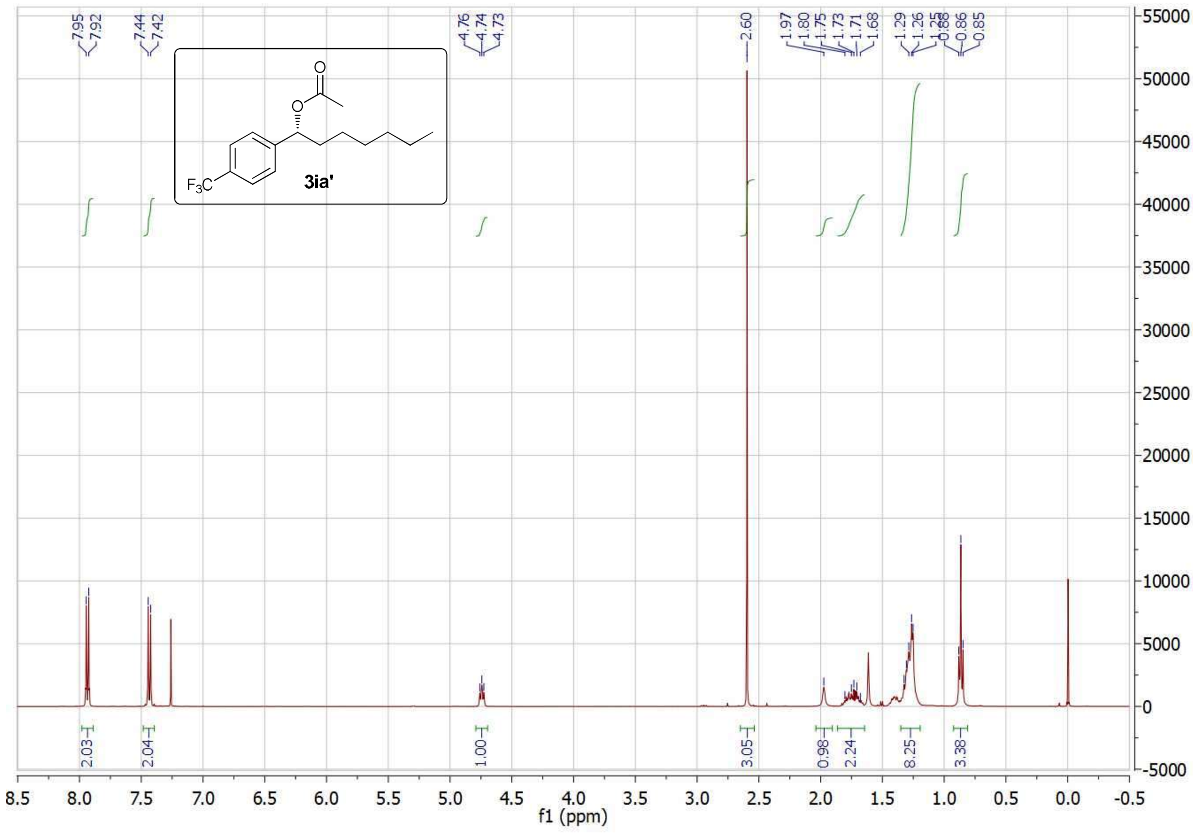

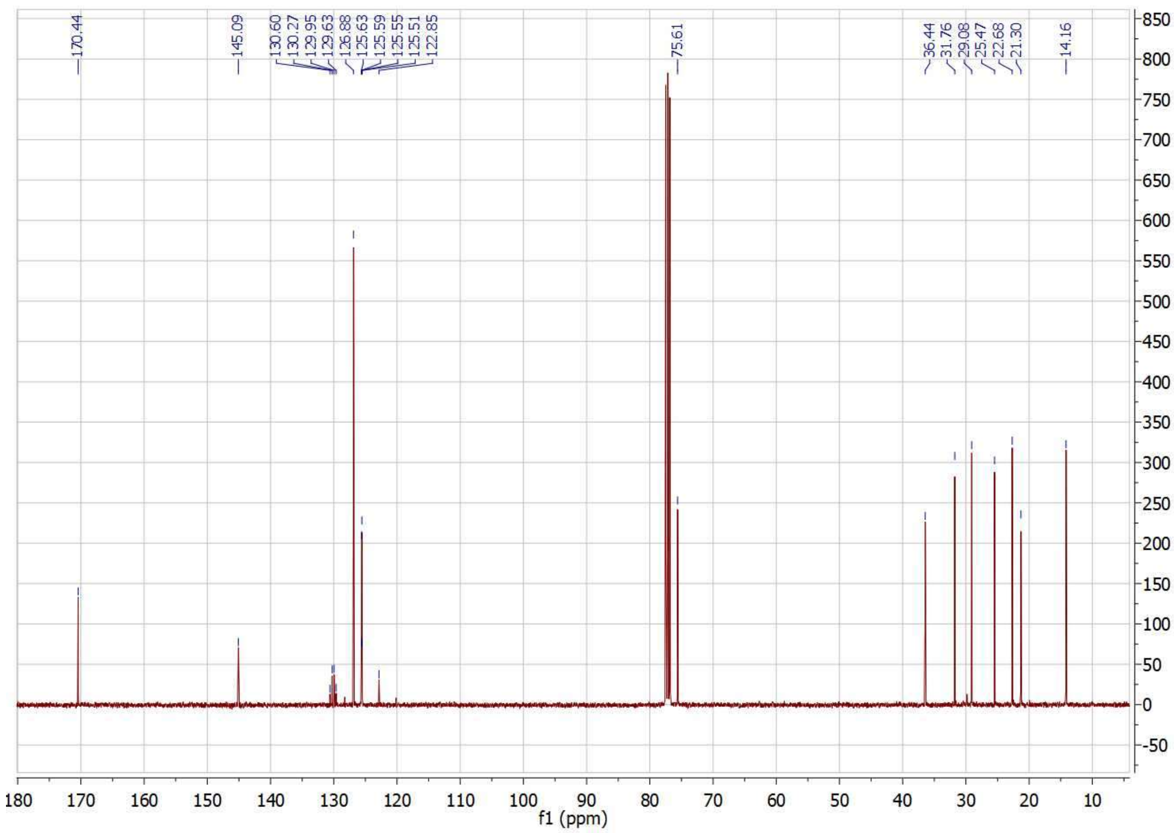

IR spectra for new compounds

(*R*)-1-m-tolylheptan-1-ol (3ca)

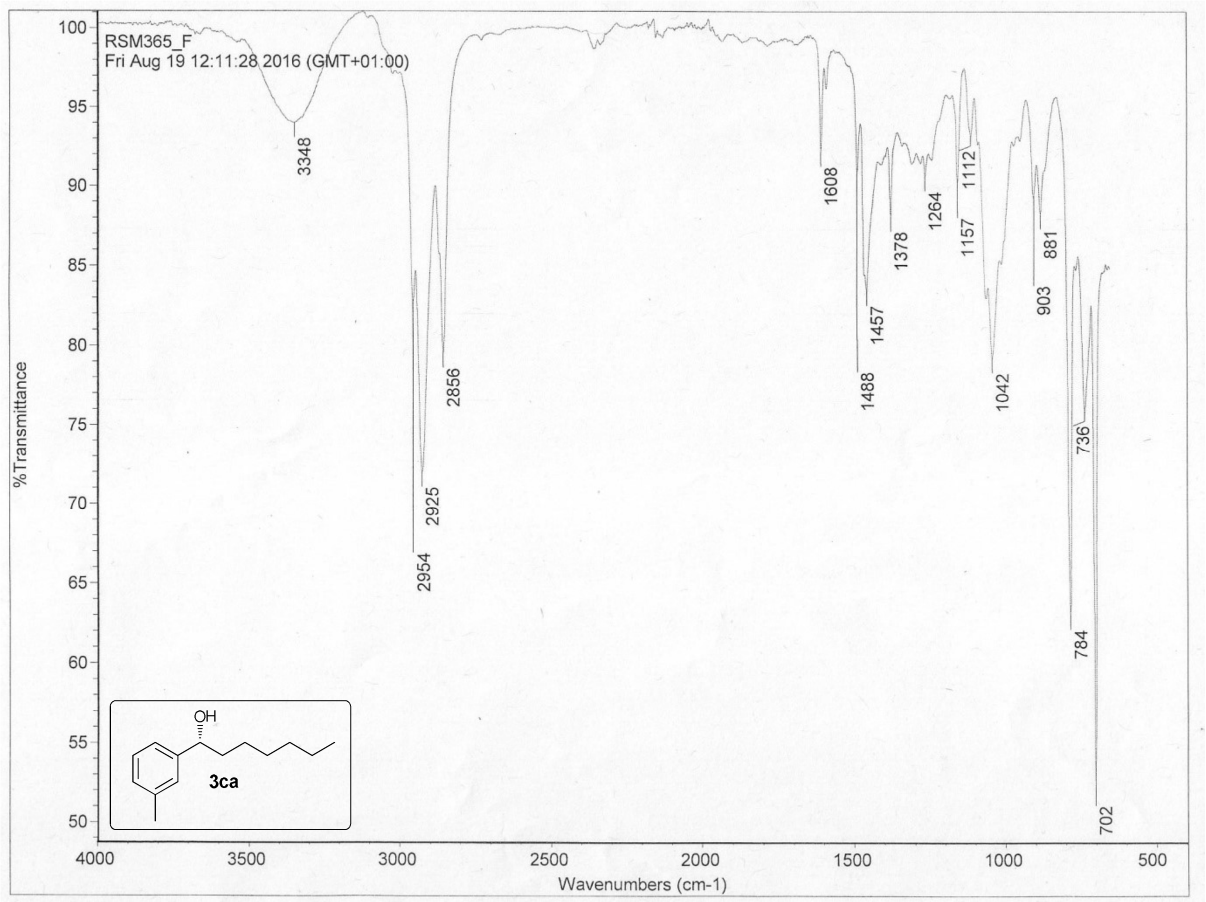

(*R*)-1-[4-(1-oxidanylheptyl)phenyl]ethanone (3ga)

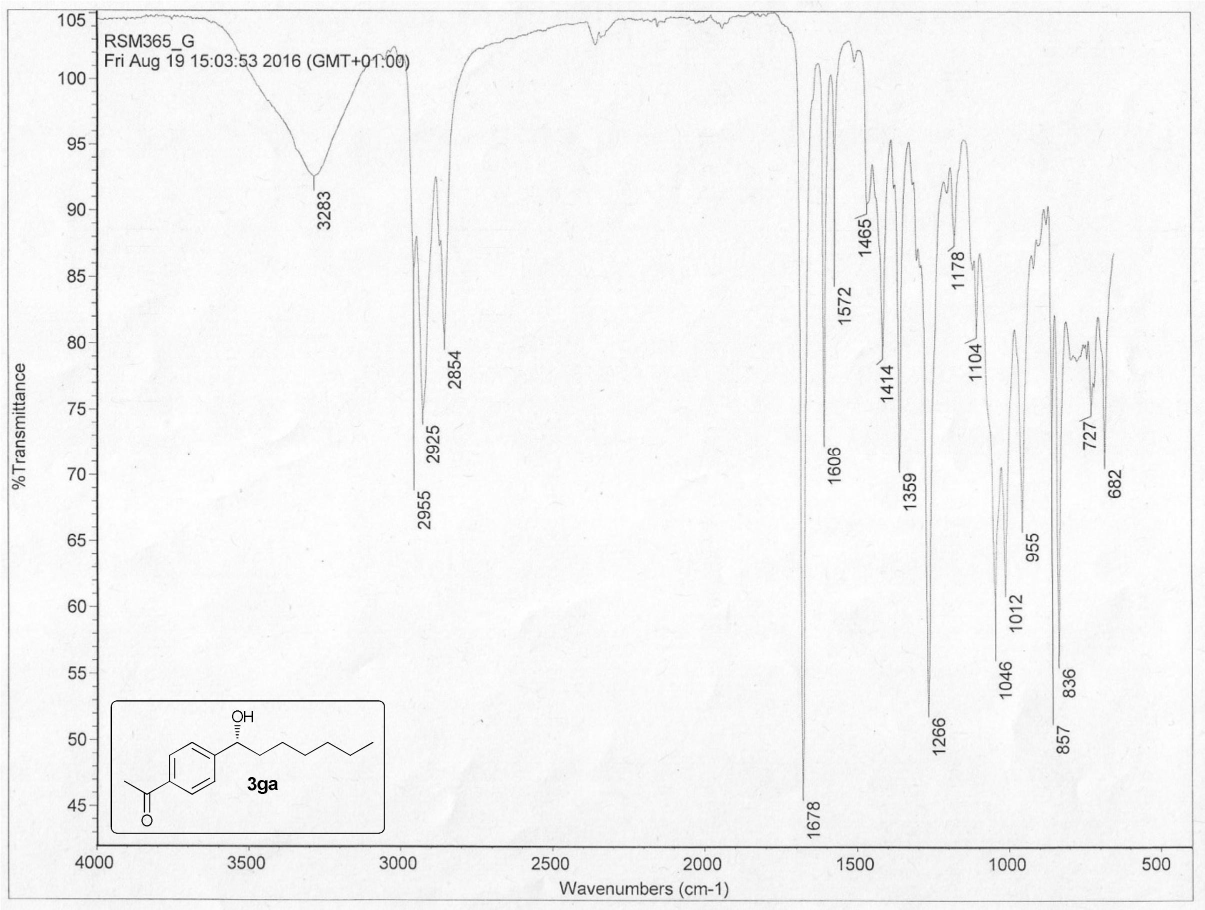

(*R*)-1-[4-(trifluoromethyl)phenyl]heptan-1-yl acetate (3ia')

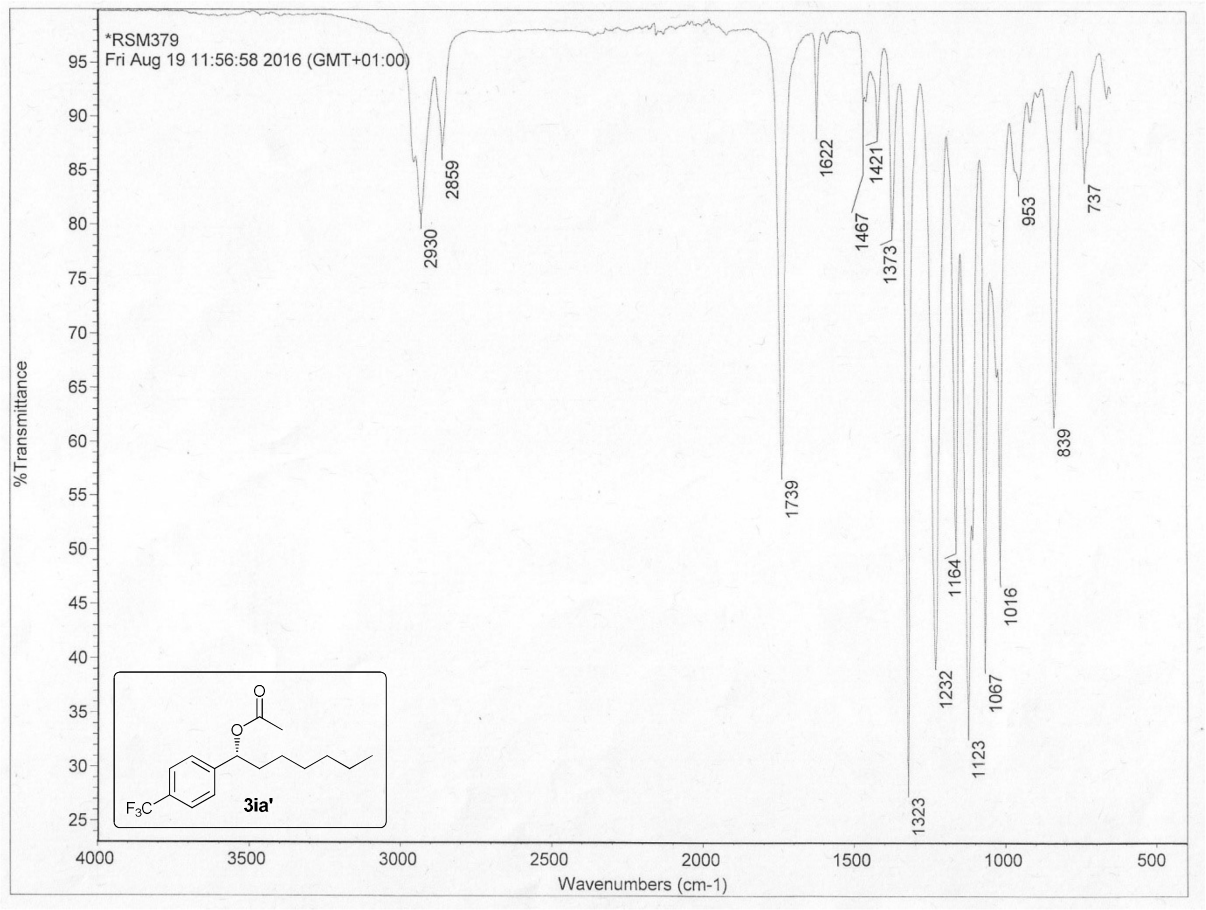

Supplement: Supplementary file 1 [file molecules-23-00961-s001.pdf]
